# Resistance to tyrosine kinase inhibitors promotes renal cancer progression through MCPIP1 tumor-suppressor downregulation and c-Met activation

**DOI:** 10.1038/s41419-022-05251-4

**Published:** 2022-09-22

**Authors:** Paulina Marona, Judyta Górka, Oliwia Kwapisz, Jolanta Jura, Janusz Rys, Robert M. Hoffman, Katarzyna Miekus

**Affiliations:** 1 Department of General Biochemistry, Faculty of Biochemistry, Biophysics and Biotechnology, Jagiellonian University, Krakow, Poland; 2 Department of Tumor Pathology, Centre of Oncology, Maria Skłodowska-Curie Memorial Institute, Cracow Branch, Garncarska 11, 31-115 Krakow, Poland; 3 AntiCancer Inc, 7917 Ostrow St, San Diego, CA 92111 USA; 4 Department of Surgery, University of California, San Diego, 9300 Campus Point Drive #7220, La Jolla, CA 92037-7220 USA

**Keywords:** Cancer therapy, Tumour heterogeneity, Tumour angiogenesis, Renal cell carcinoma

## Abstract

Tyrosine kinase inhibitors (TKIs) are the most commonly used targeted therapeutics in clear-cell renal cell carcinoma (ccRCC); however, drug resistance limits their utility and can lead to tumor “flare-up” and progression. In this study, we show that RCC resistance to sunitinib and sorafenib involves different mechanisms and leads to increased malignancy. Sunitinib decreased tumor growth and cell motility along with increased E-cadherin expression and secretion of the proangiogenic cytokines IL6 and IL8, which activated senescence in ccRCC cells and led to VE-cadherin phosphorylation, enhancing tumor angiogenesis. Sorafenib resistance increased the levels of mesenchymal markers and the secretion of MMP9, which cleaved VE-cadherin and disrupted endothelial cell integrity. Both sunitinib resistance and sorafenib resistance led to activation of the c-Met receptor IRAK1 and downregulation of the tumor suppressor MCPIP1, resulting in an increase in the metastasis of resistant cells, possibly due in part to enhanced vascularization of ccRCC. MCPIP1 overexpression partially overcame resistance to these drugs by decreasing micrometastasis and decreasing the expression of factors involved in tumorigenesis. In tumor samples from ccRCC patients, we observed a significant increase in the level of the c-Met receptor, IRAK1 and a decrease in MCPIP1 with respect to normal kidney tissue. Our results indicate separate novel mechanisms for sunitinib and sorafenib resistance, which both lead to MCPIP1 inhibition and ccRCC progression. The presented study suggests caution in the treatment of RCC with TKIs, which may lead to the unintended outcome of tumor progression.

## Introduction

Clear-cell renal cell carcinoma (ccRCC) is the most frequent (75–80%) and best-studied subtype of renal cell carcinoma (RCC) [1]. Advanced metastatic ccRCC is a lethal disease, with a 5-year survival rate of 12% [2]. Due to extensive tumor vasculature, therapy for more advanced stages of ccRCC has focused on targeting the vascular endothelial growth factor (VEGF) signaling pathway with receptor tyrosine kinase inhibitors (TKIs) or monoclonal antibodies that block VEGF [3].

The most widely used TKIs for systemic therapy in patients with RCC are sunitinib, which is approved for first-line treatment in metastatic ccRCC [4], and sorafenib, which has demonstrated progression-free survival benefits as a second-line agent [5]. Both drugs are characterized by a broad spectrum inhibition of tyrosine kinases in addition to VEGF receptor (VEGFR) kinases, including PDGF receptor beta, c-KIT, and FLT3 [1, 3]. Currently, VEGF-targeted TKI treatment in combination with PD-1/CTLA-4 blockade or anti-PD-1/PD-L1 therapy remains one of several recommended first-line treatments for patients with favorable risk metastatic RCC (mRCC) [6–8].

Although the agents have changed the therapeutic landscape for this disease, most patients initially respond to treatment but develop drug resistance and disease progression within one year [9]. A common phenomenon in RCC patients receiving TKIs is a “flare up” of tumor growth and metastasis after cessation of therapy with sunitinib or sorafenib [10, 11].

Possible mechanisms of resistance to TKIs include activation of alternative receptors or pathways, cell adaptation to a new environment, and epithelial-to-mesenchymal transition (EMT), leading to increased tumor invasiveness and dissemination [9, 12–14]. Hypoxia and hypoxia-inducible factors (HIFs) may also contribute to resistance to TKI therapy by upregulating VEGF, interleukin-6 (IL6), interleukin-8 (IL8), and hepatocyte growth factor receptor (HGFR/c-MET) [15, 16]. Moreover, growing evidence suggests that the inflammatory tumor microenvironment (TME) is a key determinant for the therapeutic efficacy of chemotherapy and immunotherapy [17]. Short-term treatment with sunitinib promotes metastasis and decreases survival time in mouse models of breast cancer [18]. However, the biological mechanism underlying TKI resistance is unclear.

In the present report, we show that although sunitinib and sorafenib have overlapping target specificities, the mechanisms of resistance are distinct. However, resistance to either drug leads to the promotion of metastasis by a common mechanism, suggesting caution in the use of TKIs for RCC, as they can lead to the unintended outcome of tumor progression.

## Materials and methods

### Patient tissue specimens

Patient tumor tissue was obtained from surgery for renal cancer in the Centre of Oncology, Maria Sklodowska-Curie Memorial Institute (Krakow Branch, Poland). The study was approved by the Hospital Institutional Review Board, and informed consent was obtained from each patient. All human tissue samples were collected using protocols approved by the Local Ethics Committee (Approval no. 68/KBL/OIL/2011). All samples were histologically evaluated by pathologists and diagnosed according to the World Health Organization classification system, specimens were divided into four groups (I–IV) according to histologic grading with the Fuhrman system. A sample of each tumor specimen was frozen in liquid nitrogen and stored at −80 °C for proteomic analysis or incubated overnight in RNAlater (Invitrogen, Waltham, MA, USA, cat. No. AM7024) at 4 °C and stored at −80 °C for RNA expression. The analysis of the MCPIP1 and c-Met protein levels and the gene expression microarray included 60 samples, 15 samples from each group (I–IV). The proteome-profiler assay included 20 samples, with 5 samples for each group. The samples were randomly chosen.

### Gene expression microarray analysis

Gene expression analysis was performed using Affymetrix HuGene ST 2.1 microarrays (Affymetrix, Santa Clara, CA, USA) on RNA samples isolated from ccRCC patient tissues as described above. For isolation of total RNA, the EURx Universal RNA Purification Kit (EURx, Gdańsk, Poland, cat no. E3598-02) was used according to the manufacturer’s protocol. The quantity of ribosomal RNA and DNA contamination was examined by electrophoresis with a 1% denaturing formaldehyde gel. The total RNA concentration was measured by a NanoDrop 1000 spectrophotometer (Thermo Fisher Scientific, Waltham, MA, USA). From each sample, 100 ng of total RNA was used to synthesize and amplify ss-cDNA, followed by fragmentation and labeling with biotin. Each step was performed according to the Affymetrix GeneChip WT PLUS Reagent Kit Manual. Next, 10 μg cRNA was hybridized for 16 h at 48 °C on the Affymetrix HuGene2.1 ST Array Strip and then washed and stained in the Affymetrix Gene Atlas Fluidics Station. Each array strip was scanned using the GeneAtlas Imaging Station. The data were normalized with Expression Console Software 1.4.1 with the RMA algorithm and analyzed using Affymetrix Transcriptome Analysis Console (TAC) Software 3.1 with one-way ANOVA between subjects (unpaired). Next, we followed the Minimum Information About a Microarray Gene Experiment (MIAME) guidelines and deposited raw and processed data in the Gene Expression Omnibus (GEO) repository under accession number GSE150404.

### Proteome-profiler assay

Proteomic analysis was performed using the Proteome Profiler Human Phospho-Kinase Array Kit (R&D Systems, Minneapolis, MN, USA, cat. no. ARY003C) according to the manufacturer’s protocol. A total of 200,000 cells were seeded in a six-well plate and cultured in the presence of sunitinib or sorafenib for one week in six-well plates. Next, the cells were washed with ice-cold Phosphate Buffered Saline (PBS, Lonza, Basel, Switzerland, cat no. BE17-513F), harvested, and lysed with lysis buffer 6 (R&D Systems, provided with the kit) for 30 min. Tumor tissue samples were homogenized with lysis buffer 6. The lysates were then spun for 5 min at 15,000x*g* and 4 °C. Next, 600 μg of each lysate was taken per array set. Chemiluminescence was detected after 10 min of incubation with Chemi Reagent Mix (R&D Systems, provided with the kit) using a ChemiDoc system (Bio-Rad, Hercules, CA, USA). The densitometric values of each set of dots were measured using Image Lab 5.2.1 software (Bio-Rad).

### Cell culture

The human ccRCC cell line Caki-2 was obtained from Sigma-Aldrich, St. Louis, MO, USA (cat. no. 93120819). Caki-1 cells were obtained from ATCC (cat no. HTB-46, Manassas, VA, USA). HUVECs were kindly provided by the Department of Transplantology, Medical College (Krakow, Poland). The cells in the initial vials were expanded and cryopreserved, and cells were propagated with less than fifteen consecutive passages. All cell lines were routinely tested for mycoplasma by PCR every three months. The Caki-2 and Caki-1 cell lines were cultured in Eagle’s minimal essential medium (EMEM; Lonza, Basel, Switzerland) supplemented with 10% fetal bovine serum (FBS). HUVECs were cultured in endothelial basal medium (EBM-2, Lonza) supplemented with EGM-2 MV SingleQuots (Lonza). All cell lines were cultured at 37 °C in a humidified incubator in a 5% CO_2_ atmosphere. To collect conditioned medium from the Caki-1 and Caki-2 cell lines, cells were cultured for 7 days in the presence of drugs, followed by 24 h in medium supplemented with only 0.5% BSA (BioShop, Burlington, Ontario, Canada); the culture medium was then collected for further analysis.

### Authentication

All cell lines were authenticated by GenMed (Poznań, Poland) in August 2019 using STR DNA profiling methods within the loci of D8S1179, D21S11, D3S1358, TH01, D16S539, D2S1338, D19S433, vWA, D18S51, D10S1248, D22S1045, D2S441, D1S1656, D12S391, FGA, and AMEL. Raw and processed data from the microarray analysis are deposited in the Gene Expression Omnibus repository: (accession number: GSE150404).

### In vitro drug-sensitivity testing

Sunitinib and sorafenib (both from LC Laboratories, Woburn, MA, USA cat. no. S88031G and S85991G), SU11274 (c-Met inhibitor; Selleckchem, Houston, TX, USA, cat. no. S1080), and U0126 (ERK inhibitor; Sigma-Aldrich, Saint-Louis, Missouri, USA, cat no. U120), stock solutions were prepared in DMSO (BioShop, Burlington, Ontario, Canada). Next, working solutions were prepared in culture medium (SU11274—2.5 µM, sunitinib—5 µM, sorafenib—2.5 µM, U0126—10 µM). For long-time testing, the medium was changed every second day and replaced with fresh medium with newly added drugs. Caki-1 pLIX-PURO and pLIX-MCPIP1 were treated 48 h with doxycycline followed by 48 h with doxycycline and drugs. DMSO was used as a control at a final concentration of 0.1% (v/v) in culture medium.

### Stable transduction of MCPIP1 with viral vectors

For stable overexpression of MCPIP1, a doxycycline-dependent lentiviral TetON system was used (pLIX-MCPIP1, pLIX-PURO and the mutant form pLIX D141N). A GFP-expressing lentiviral vector was used. Briefly, ccRCC cells were plated at 50% confluency in six-well plates. Viral vectors were added at a multiplicity of infection (MOI) of 50 with 6 mg/mL polybrene (Millipore). To increase the transduction efficiency after 24 h, the process was repeated. Cells were incubated with viruses for 24 h, and then the medium was changed. After an additional 24 h, 1 μg/mL puromycin (InvivoGen, San Diego, CA, USA) was added to start selection. To induce MCPIP1 overexpression in the TetON system, 1 μg/mL doxycycline (BioShop) was added for 24–48 h.

### RNA isolation and quantitative RT-PCR

Total RNA was isolated from cultured cells using the Universal RNA Purification Kit (EURx, Gdańsk, Poland, cat no. E3598-02). RNA was isolated from intact mouse lungs using fenozol (phenol–chloroform extraction, A&A Biotechnology, Gdańsk, Poland, cat. no. 203-100). The quantity of ribosomal RNA and the presence of DNA contamination were examined using electrophoresis with a 1% denaturing formaldehyde gel. The concentration of total RNA was assessed using a NanoDrop 2000 spectrophotometer (Thermo Fisher Scientific). Reverse transcription was performed using 1 mg of total RNA, oligo(dT) 15 primer (1 μg/μl, Promega, Madison, WI, USA, cat. no. C1101), dNTPs (10 mM, Promega, cat. no. U1330) and M-MLV reverse transcriptase (Promega, cat. no. M1701). Real-time PCR was carried out using SYBRGreen Master Mix (A&A Biotechnology, cat. no. 2008-1000A) and QuantStudio 3 (Applied Biosystems, Waltham, MA, USA). For the examination of mouse lung metastasis, specific probes for human GAPDH and mouse GAPDH (Life Technologies, Carlsbad, CA, USA, cat. no. 4333764F, 4352932E) were used with Taq PCR Master Mix (EURx, cat. no. E2520-01). Gene expression was normalized to the expression of elongation factor-2 (EF2). The mRNA level in each sample was analyzed in duplicate. The relative levels of transcripts were quantified by the ΔΔCt method. The sequences of primers (Sigma-Aldrich) and annealing temperatures are listed in Supplementary Table S1.

### Western blot analysis

Cultured cells were washed with ice-cold PBS (Lonza), harvested, and lysed with lysis buffer 6 (R&D, cat. no. 895561) for 30 min. The lysates were then centrifuged for 5 min at 15,000 x *g* and 4 °C. SDS-PAGE was conducted with a 10% polyacrylamide gel. After wet transfer to polyvinylidene difluoride membranes (Millipore, cat. no. IPVH00010), the membranes were blocked in 3% BSA in Tris-buffered saline with 0.1% Tween 20 (Sigma-Aldrich). Next, the membranes were incubated with primary antibodies at 4 °C overnight with gentle agitation. On the following day, the membranes were washed three times for 10 min with TBS with 0.1% Tween 20 and incubated with a secondary antibody for 1 h at room temperature (RT) with gentle rocking. Chemiluminescence was detected after a 5-min incubation with Immobilon Western HRP substrate (Millipore, cat no. WBKLS0050) using a ChemiDoc system (Bio-Rad). All antibodies and dilutions are listed in Supplementary Table S2.

### MTT assay

Five thousand cells per well were seeded in 96-well plates. After 24 h, the cells were stimulated with drugs as described earlier. MTT solution (0.5 mg/mL, Sigma-Aldrich, cat. no. M5655) was added for 2 h. The reaction was stopped by adding 5 mmol/L HCl (POCH, Gliwice, Poland) to isopropanol (Chempur, Piekary Śląskie, Poland), and the plate was shaken for 30 min to dissolve the formazan crystals. The absorbance was measured at 570 nm with a reference wavelength of 500 nm using a Tecan Spectra Fluor Plus microplate reader (Tecan Group Ltd., Mannedorf, Switzerland). Measurements were performed 24 h, 48 h, 96 h, and 7 days after the first stimulation. Three independent experiments were performed in triplicate.

### Crystal violet staining

Cultured cells were washed with PBS and fixed with 100% methanol for 5 min at room temperature. Next, the cells were stained with a 20% crystal violet solution in methanol for 20 min at room temperature. After three washes with PBS, the cells were air dried. All images were acquired using a Nikon Eclipse Ti-S microscope with ×10, ×20, and ×40 objectives (Nikon, Minato, Tokyo, Japan). For analysis and measurement of cell morphology and area, ImageJ software was used (National Institutes of Health, Bethesda, MD, USA).

### Cell-cycle analysis

After one week of exposure to drugs, cells were harvested with Accutase (BioLegend, San Diego, CA USA, cat. no. 423201) and counted. A total of 100,000 cells were fixed with ice-cold 70% ethanol for 30 min at 4 °C. Then, the cells were centrifuged for 3 min at 3000 rpm, washed with PBS, and again centrifuged under the same conditions. Next, the cells were resuspended in 250 μl of staining solution (1 µl of 50 mg/ml propidium iodine (Life Technologies, V35118) and 25 µl of 100 mg/ml RNAse A (Sigma-Aldrich, cat. no. R4642) in 10 ml of PBS) and incubated for 30 min at 37 °C. Measurements were performed using an Attune NxT acoustic focusing cytometer and analyzed with Attune software v2.4 (Thermo Fisher Scientific).

### Immunofluorescence staining of cultured cells

Cells were plated on glass coverslips in 24-well culture plates at a density of 50,000 cells per well and grown in the presence of drugs (Caki-1) or to full confluence (HUVECs). The cells were washed with PBS, fixed for 15 min in 4% paraformaldehyde (Chempur) at room temperature, and then washed three times with PBS. Next, the cells were permeabilized and blocked with blocking buffer (1% BSA in PBS with 0.3% Triton X-100 (Sigma-Aldrich)) at room temperature for 1 h. Incubation with primary antibodies against phospho c-Met (1:200; Cell Signaling, Danvers, MA, USA); E-cadherin (1:200; Abcam, Cambridge, UK); β-catenin (1:200; Cell Signaling; cat. no. 19807S); and VE-cadherin (1:100; Abcam) in PBS with 1% BSA was performed overnight at 4 °C. On the following day, secondary antibodies conjugated with Alexa Fluor 546, Alexa Fluor 488 or Alexa Fluor 647 (1:1000; Thermo Fisher Scientific) or rhodamine phalloidin reagent (1:1000; Abcam, ab235138) and Hoechst nuclear dye (Invitrogen, Waltham, MA, USA, cat. no H3570) were added and incubated for 1 h in the dark at room temperature. All samples were mounted with Dako Mounting Medium (Agilent Technologies, Santa Clara, CA, USA, cat. no. CS70330-2) and sealed with nail polish. Images were acquired with a Leica DM6 B fluorescence microscope (Leica Microsystems, Wetzlar, Germany) with ×10 and ×20 dry objectives and a ×63 oil immersion objective with Leica LAS X image acquisition software.

### Clonogenic assay

After one week of exposure to drugs, cells were trypsinized and counted, and 1000 cells that survived drug exposure were seeded in six-well plates. After 2 weeks, clones were stained with crystal violet, and images of the wells were acquired with a ChemiDoc system (Bio-Rad).

### Migration/wound-healing assay

Cells were grown in 24-well plates to full confluence in the presence of drugs. Immediately before the experiment, 10 mmol/L hydroxyurea (Sigma-Aldrich, cat. no. H8627) was added to the culture medium to inhibit cell proliferation. A scratch was made with a small pipette tip. Then, the plate was transferred to a microscope culture chamber at 37 °C and 5% CO_2_. Images were acquired every 10 min for 16 h using a Leica DMI6000B inverted widefield fluorescence microscope (Leica Microsystems). All images were recorded using a ×10 dry objective with Leica LAS X image acquisition software and analyzed with Hiro v. 1.0.0.4 software.

### SA-β-gal staining for senescence

Cells were seeded on coverslips in 24-well culture plates. After one week of drug exposure, the cells were stained for SA-β-gal with a staining kit (Cell Signaling, cat. no. 9860S) as described by the manufacturer. Briefly, the cells were fixed with Fixative Solution for 15 min at room temperature, then β-gal Staining Solution was added, and the plates were incubated at 37 °C overnight in a dry incubator. On the next day, the samples were mounted with Glycergel Mounting Medium (Agilent Technologies, cat. no. C0563). All images were acquired with a Leica DM6 B fluorescence microscope (Leica Microsystems) with a ×10 dry objective and Leica LAS X image acquisition software. Quantification was performed manually. First, on each image, blue cells (positive for SA β-gal) were count, followed by counting all cells. Four images were analyzed per treatment, per experiment. Three independent experiments were performed.

### Mouse experiments

Mouse experiments were conducted in accordance with protocols approved by the Institutional Animal Care and Use Committee: II Local Ethics Committee of the Institute of Pharmacology Polish Academy of Sciences (Approval no. 20/2017 and 53/2019). Mice were handled according to the regulations of national and local animal welfare bodies. Six-week-old female NOD-SCID mice (Charles River Laboratory, Wilmington, MA, USA) were kept under specific pathogen-free (SPF) conditions, with water and food provided ad libitum. Caki-1 GFP cells were incubated 5 weeks with DMSO (control), sunitinib or sorafenib. Resistant Caki-1 GFP cells were mixed with wild-type Caki-1 cells in 1:1 ratio (total number of cells 2 × 10^6^ cells) before subcutaneous injection. Caki-1 pLIX-PURO and Caki-1 pLIX-MCPIP1 cells were incubated 5 weeks with DMSO (control), sunitinib or sorafenib. Resistant cells were injected subcutaneously as a cell suspension (2 × 10^6^ cells in PBS). Mice injected with resistant Caki-1 pLIX-PURO or pLIX-MCPIP1 drank water with doxycycline (200 mg/L) to induce MCPIP1 overexpression. Tumor growth was monitored for 6 weeks. Tumor volume was estimated using caliper measurements, according to the formula: volume = width × depth^2^ of the tumor. After tumor and lung excision, RNA isolation and histologic analysis were performed. To obtain frozen sections, tissues were prefixed in pure buffered formaldehyde (Chempur) for 3 h, washed in PBS, incubated for 12 h in 30% sucrose at 4 °C and embedded in OCT (VWR Chemicals, Radnor, PA, USA). Then, nine-micron-thick sections were prepared and analyzed for fluorescence signals from GFP-positive cells. All images were acquired with a Leica DM6 B fluorescence microscope (Leica Microsystems) with ×5 and ×20 dry objectives and Leica LAS X image acquisition software.

### ELISA

Human DuoSet ELISA kits for IL8 and IL6 (R&D Systems, cat no. DY208, DY206) were used to evaluate the levels of secreted proteins in conditioned medium or mouse plasma, according to the manufacturer’s protocols. The absorbance was measured at 450 nm with a reference wavelength of 540 nm using a Tecan Spectre Fluor Plus microplate reader. Three independent experiments were performed in triplicate for conditioned medium experiments and without replicates for each mouse sample.

### Immunohistochemical (IHC) staining

Fresh tissues were prefixed in pure buffered formaldehyde (Chempur), washed in PBS, incubated for 12 h in 30% sucrose at 4 °C and embedded in OCT (VWR Chemicals). Then, 9-μm slides were cut on a cryostat (Leica) and placed on poly-l-lysine-coated slides. Next, the sections were permeabilized (0.1% Triton X-100 in PBS) and blocked in blocking buffer (5% BSA or 2% powdered milk in PBS) at room temperature for 1 h. Samples were stained with a primary rat anti-mouse CD31 antibody or an isotype control (1:50, BD Pharmingen, San Jose, CA, USA, 4163758 and 4234535); anti-selectin P (SelP) antibody (10 µg/ml, R&D Systems, AF737) or anti-ICAM-1 antibody (1:100, eBioscience, Affymetrix, USA, 14-0542-82) in 1% BSA in PBS. On the following day, the slides were washed with PBS and incubated for 1 h at room temperature with secondary antibodies conjugated with Alexa Fluor 594, Alexa Fluor 568 or Alexa Fluor 546 (1:1000; Thermo Fisher Scientific) and Hoechst nuclear stain. The slides were mounted with Dako Fluorescent Mounting Medium (Agilent Technologies, cat. no. CS70330-2). Images were acquired with a Leica DM6 B fluorescence microscope with a ×5 or ×20 dry objective and Leica LAS X image acquisition software. IHC evaluation was also performed using primary rabbit polyclonal anti-CD31 antibody (1:50, Abcam) and EnVision Detection Systems Peroxidase/DAB, Rabbit/Mouse (DakoCytomation) to visualize tumor vascularization.

### Statistical analysis

All in vitro experiments were conducted at least three times independently. The number of animals or patient samples is indicated in the figure legends. All results are shown as the mean ± SD, except for animal studies where results are presented as the mean ± SEM. For graph preparation and statistical analysis, GraphPad Prism 7 (San Diego, CA, USA) was used, except for the circularity/area graph, which was prepared using Origin 2019b software (OriginLab, Northampton, MA, USA). For comparison of two groups, the Student’s *t* test was used. For comparisons of three or more groups, one-way or two-way ANOVA with the post hoc Tukey multiple comparisons test was used. *P* values are marked with asterisks in graphs (*P < 0.05; **P < 0.01; ***P < 0.001; ****P < 0.0001 versus the control).

### Ethics statement

The animal experiments were approved by the II Local Ethics Committee of the Institute of Pharmacology Polish Academy of Sciences (Approval nos. 20/2017 and 53/2019). Biopsies of renal tumors were obtained from patients surgically treated for renal cancer in the Centre of Oncology, Maria Sklodowska-Curie Memorial Institute, Cracow Branch, under the supervision of the Local Ethics Committee (Approval no. 68/KBL/OIL/2011).

## Results

### Sunitinib and sorafenib differentially affect the viability, cell morphology, and kinase activity of ccRCC cells in vitro

**Fig. 1:**
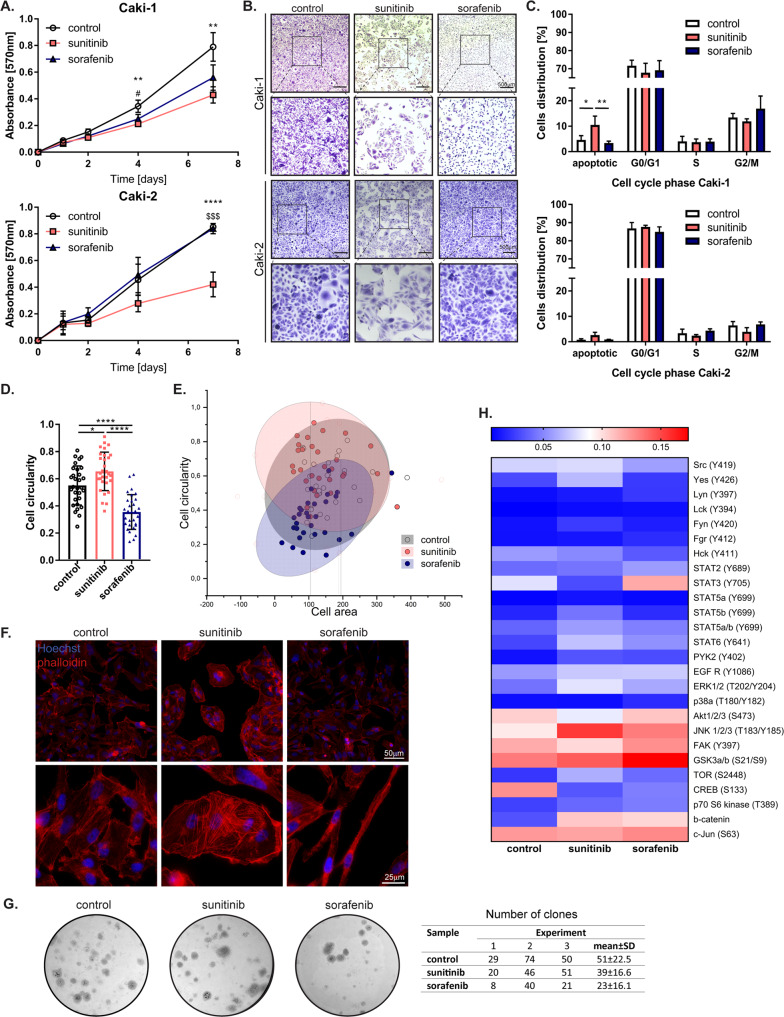
Effect of drugs on ccRCC viability, cell cycle, and morphology. **A** MTT assay on Caki-1 and Caki-2 cells treated 7 days with DMSO (control), sunitinib or sorafenib. The results are presented as the triplicate mean ± SD of three independent experiments. *P* values were estimated using one-way ANOVA with post hoc Tukey’s multiple comparison test. ^#^*P* < 0.05 control vs sorafenib; **P < 0.01; ****P < 0,0001 control vs sunitinib; ^$$$^*P* < 0,001 sunitinib vs sorafenib. **B** Representative images of crystal violet stained Caki-1 and Caki-2 cells after 96-h stimulation with drugs. **C** Calculation of Caki-1 cells distribution between cell-cycle phases. The results are presented as the mean ± SD of three independent experiments. *P* values were estimated using two-way ANOVA with post hoc Tukey’s multiple comparison test. **D** Quantification of Caki-1 cell shape after 7 days of treatment. *N* = 30 per group. The results are presented as the means ± SD. *P* values were estimated using one-way ANOVA with post hoc Tukey’s multiple comparison test. **E** Correlation between cell shape and cell area presented with 2D Confidence Ellipse (confidence level 95%). *N* = 30 per group. **F** Representative images after immunofluorescence staining for phalloidin and Hoechst, of Caki-1 cells after 7 days stimulation with DMSO, sunitinib or sorafenib. **G** Representative images of clones formed by resistant Caki-1 cells, stained with crystal violet. Table with the number of clones in three independent experiments. All clones were counted from each cell-culture well. The final result is presented in the last column as mean ± SD. **H** Heatmap representing mean of densitometric values from proteome-profiler analysis. All experiments were performed three times. *P < 0.05; **P < 0.01; ***P < 0.001; ****P < 0.0001.

Sunitinib and sorafenib are multitarget drugs that are frequently used to treat patients with ccRCC. However, after some time of treatment, most patients develop strong resistance to these molecules, and the mechanism underlying this resistance is not fully understood [9]. We observed that ccRCC cells (Caki-1 and Caki-2) treated with sunitinib showed significantly decreased viability compared to control cells. Sorafenib-treated Caki-1 cells also had decreased viability (Fig. 1A, B and Supplementary Fig. 1). In our study, the IC50 of sunitinib in Caki cells was approximately 5 μM, and that of sorafenib was 8 μM. However, due to the observed phenotypic changes, the lowest dose of sorafenib (2.5 μM) was used for further experiments (Supplementary Fig. 1). Flow cytometric analysis showed that sunitinib induced a high rate of apoptosis, whereas sorafenib treatment induced a high rate of G2/M phase arrest, which explained why the level of proliferation was similar to that of cells under control conditions (Fig. 1A–C and Supplementary Fig. 2A–D). After 1 week of drug treatment, we also observed phenotypic changes (Fig. 1D–F). We found that sunitinib-resistant cells were mostly spherical. In contrast, the sorafenib-resistant cells were small and spindle-shaped (Fig. 1D–F). Phalloidin staining demonstrated that sunitinib-resistant cells started to form colonies. Sorafenib-resistant cells had a spindle and a mesenchymal phenotype, which was not observed for control, untreated cells (Fig. 1F). In addition, we observed a more than twofold decrease in the number of drug-resistant colonies established by sorafenib-resistant cells compared to cells not previously treated with sorafenib (Fig. 1G). To further examine the influence of drug treatment on tumor cells, we performed proteomic profiling after 1 week of constant drug treatment. We found upregulation of the phosphorylated forms of STAT3, EGFR, JNK, FAK and c-Jun and of total β-catenin in sunitinib- and sorafenib-resistant cells (Fig. 1H). These data indicate that both sunitinib and sorafenib affect proliferation and the cell cycle and modify signal transduction pathways and that ccRCC cells continue to proliferate and show resistance in the presence of these drugs.

### Sorafenib-resistant ccRCC cells had increased cell migration ability and mesenchymal phenotype marker expression

**Fig. 2:**
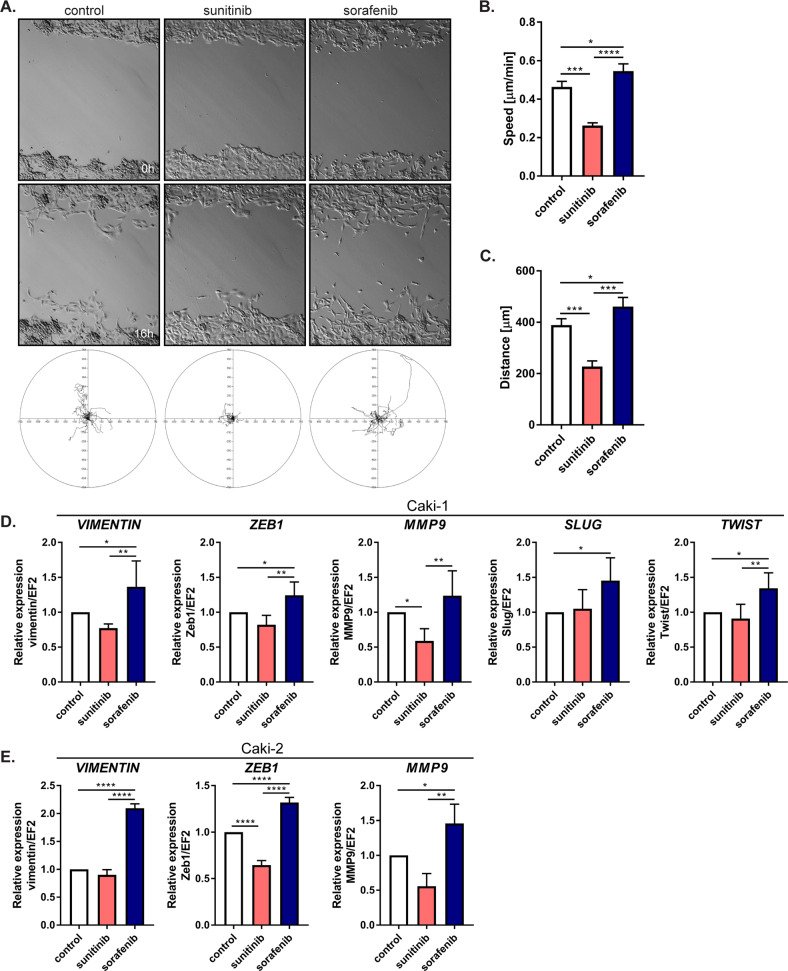
Effect of sorafenib on EMT. **A** Representative images after 0 and 16 h (upper panel) of migratory paths during a 16-h time-lapse recording, *N* = 18 cells for each treatment (lower panel). **B** Quantification of the distance traveled by Caki-1 cells during the 16-h experiment. **C** Quantification of the speed of Caki-1 cells during the 16-h experiment. The results are presented as the mean ± SD of three independent experiments, with the number of cells analyzed for each cell line, *N* = 55. **D** mRNA level of EMT markers in Caki-1 cells after 24 h treatment with drugs, quantified with real-time PCR. **E** mRNA level of EMT markers in Caki-2 cells after 24 h treatment with drugs, quantified with real-time PCR. The results are presented as the mean ± SD of three independent experiments. *P* values were estimated using One-way ANOVA with post hoc Tukey’s multiple comparison test. *P < 0.05; **P < 0.01; ***P < 0.001; ****P < 0.0001.

As we observed that sunitinib and sorafenib treatments changed cell morphology, we wondered whether prolonged treatment with these drugs induces the acquisition of mesenchymal features, including migration ability and increased expression of mesenchymal markers. We found that sorafenib-resistant cells had high migration activity, while migration was strongly suppressed in sunitinib-treated cells compared to control cells, as shown in an in vitro wound-healing assay (Fig. 2A–C). An analysis of the individual tracks of more than 50 cells per group showed that the values for distance and speed were highest for sorafenib-resistant cells and lowest for sunitinib-resistant cells (both compared to control, nontreated cells) (Fig. 2B, C). To identify the mechanism of cell migration activation, we evaluated changes in the levels of various epithelial–mesenchymal transition (EMT) markers. We observed significant increases in *vimentin, MMP9, ZEB1, SLUG,* and *TWIST* expression in sorafenib-treated cells (Fig. 2D, E), whereas sunitinib treatment decreased migration activity and the expression of mesenchymal markers in Caki-1 and Caki-2 cells. These results show that the processes underlying resistance to sunitinib or sorafenib have different molecular backgrounds and promote different cell behaviors.

### Sunitinib treatment induces senescence in ccRCC cells

**Fig. 3:**
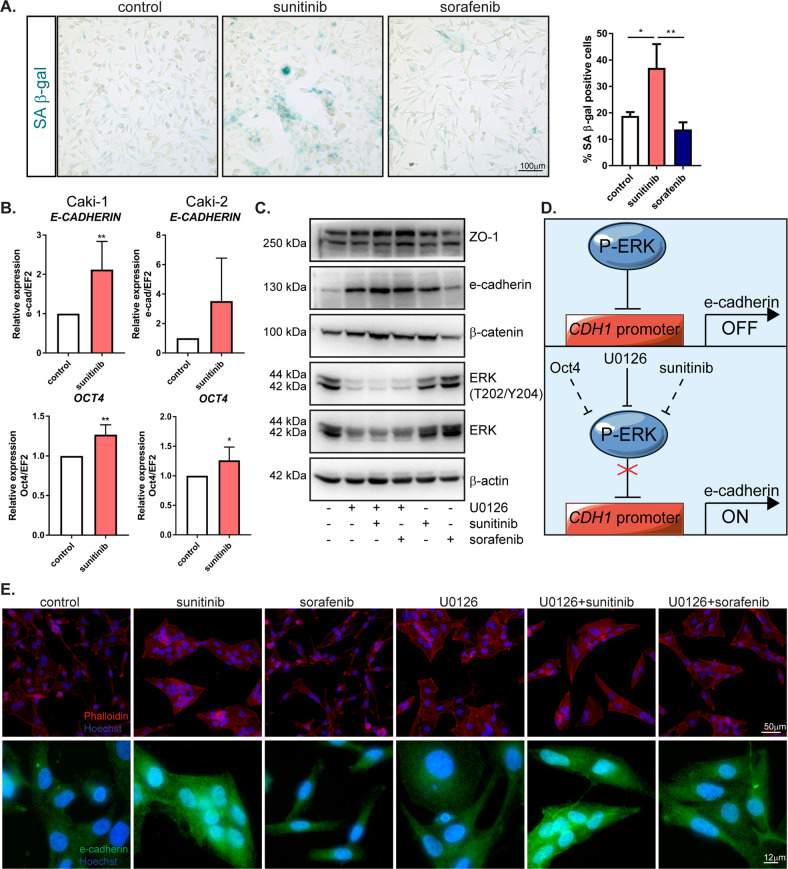
Mechanism of sunitinib resistance. **A** Representative images after SA β-gal staining on Caki-1 cells after 1 week of drug treatment. The graph represents the quantification of SA β-gal positive cells to all cells. **B** mRNA level of *E-CADHERIN* and *OCT4* in Caki-1 and Caki-2 cells after 24 h treatment with drugs, quantified with real-time PCR. The results are presented as the mean ± SD of three independent experiments. *P* values were estimated using one-way ANOVA with post hoc Tukey’s multiple comparison test. *P < 0.05; **P < 0.01; ***P < 0.001. **C** Representative western blot of Caki-1 cells after 24-h treatment with the ERK inhibitor U0126 or in combination with sunitinib or sorafenib. β-actin was used as a loading control. **D** Schematic representation of the role of ERK in E-cadherin expression. ERK phosphorylation inhibits the CDH1 promoter which leads to decreased E-cadherin expression. ERK activity might be blocked directly by specific inhibitor U0126 or indirectly by Oct4 or sunitinib. **E** Representative images after immunofluorescence staining of phalloidin and E-cadherin on Caki-1 cells after 24-h treatment with U0126, sunitinib or sorafenib. Hoechst was used to visualize nuclei. Experiments were performed at least three times.

A decrease in the ability to proliferate, changes in the cell cycle and loss of cellular motility may be signs of cell senescence [19]. In sunitinib-resistant cells, in addition to a reduction in the proliferation rate (Fig. 1A), an increased number of apoptotic cells (Fig. 1C) and a decrease in migratory potential (Fig. 2A–C), we observed a strong increase in senescence-associated β-galactosidase (SA-β-gal) activity compared to that in the control group (Fig. 3A). In contrast, no increase in β-gal activity in sorafenib-resistant cells was observed (Fig. 3A). Premature senescence in culture may be a result of mesenchymal-to-epithelial transition (MET), the reverse process of EMT characterized by the repression of mesenchymal genes, and the activation of epithelial genes encoding epithelial cell junction proteins, such as E-cadherin [20–22]. Our results showed that sunitinib treatment led to a significant decrease in the expression of *TWIST* and *ZEB1*, regulators of the level of E-cadherin (Fig. 2D, E), and an increase in E-cadherin (Fig. 3B). Sunitinib treatment also induced high expression of mRNA transcripts for octamer-binding transcription factor 4 (*OCT4*), a regulator of cancer stemness [23] (Fig. 3B). Moreover, we observed that sunitinib treatment decreased the levels of total and phosphorylated ERK (Fig. 3C). ERK was shown to regulate E-cadherin [24, 25]. We found that administration of the ERK inhibitor U0126 alone or combined with sunitinib or sorafenib upregulated the epithelial markers E-cadherin, ZO-1 and β-catenin, similar to treatment with sunitinib alone (Fig. 3C, D and Supplementary Fig. 3). We also observed that after ERK inhibition, epithelial morphology was restored, and cells started to form colonies, which was similar to the features of sunitinib-treated cells (Fig. 3E). We concluded that this undifferentiated, senescent state with high E-cadherin and Oct4 levels may be, at least in this model, a mechanism that partially explains of sunitinib resistance.

### Sunitinib and sorafenib resistance promotes tumor growth, metastatic progression, and tumor vascularization

**Fig. 4:**
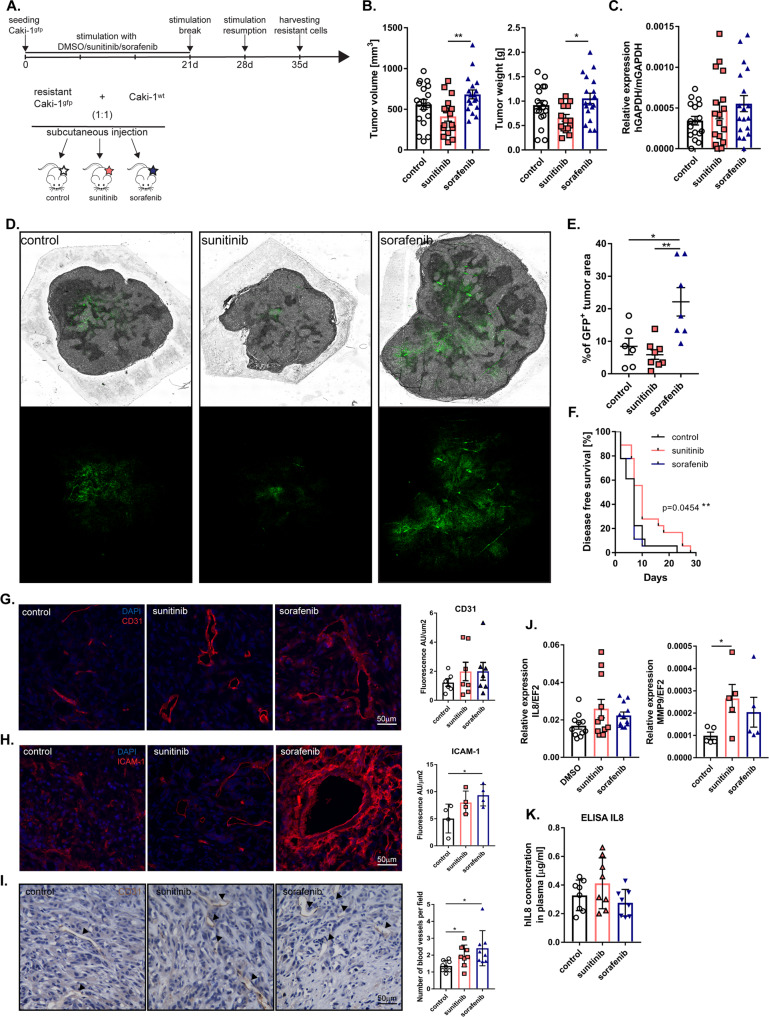
Effect of sunitinib and sorafenib resistance on tumor growth, metastasis, and angiogenesis. **A** Schematic representation of in vivo experiments. GFP-positive Caki-1 cells were treated with drugs constantly for 3 weeks. After a 7-day break to increase the cell number, cells were treated for the next 7 days. Next, resistant cells were harvested and mixed with wild-type Caki-1 in a 1:1 ratio and injected subcutaneously into NOD-SCID mice. **B** Effect of drug resistance on tumor volume (left graph) and tumor weight (right graph). control, *N* = 18; sunitinib, *N* = 16; sorafenib, *N* = 18. **C** mRNA analysis of lung metastasis using real-time PCR. *N* = 17 for each group except sorafenib, *N* = 18. **D** Representative merged images of bright-field and GFP fluorescence (upper panel) or only GFP fluorescence (lower panel) of tumor sections **E** Percentage quantification of GFP-positive tumor area to total tumor area in tumor sections, *N* = 6 control; *N* = 8 sunitinib; *N* = 7 sorafenib. **F** Percentage of tumor-free mice after cancer-cell injection. *P* value summary ** calculated with Log-rank (Mantel–Cox) test, *N* = 18 per group. **G**, **H** Representative images after immunofluorescence staining of CD31 (**G**) and ICAM-1 (**H**). **I** CD31 IHC staining of tumor sections and quantification of functional vessels with a visible lumen. **J** mRNA level of *MMP9* and *IL8* in tumors, quantified with real-time PCR. *IL8*, *N* = 10 per group, *MMP9*, *N* = 5. **K** The level of secreted IL8 in mouse plasma obtained using ELISA, *N* = 8 per group. Tumors were collected 6 weeks after s.c. injection of RCC cells. The results are presented as the mean ± SEM. *P* values were estimated using One-way ANOVA with post hoc Tukey’s multiple comparison test, except *MMP9* where the Student’s *t* test was used. *P < 0.05; **P < 0.01.

To investigate the effect of acquired resistance to sunitinib or sorafenib on tumor growth and metastasis, we transduced Caki-1 cells with a GFP lentiviral vector and treated the cells with sunitinib or sorafenib constantly for 3 weeks. Then, after a 7-day break to expand the cells, the cells were treated with drugs for an additional 7 days. After this time, to model heterogeneous tumor cell populations in vivo, we combined drug-resistant GFP^+^ cells with wild-type Caki-1 cells at a 1:1 ratio and injected them into NOD-SCID mice to compare their capability to form tumors (Fig. 4A). After 6 weeks, we observed higher tumor volume and tumor weight in mice bearing tumors derived from sorafenib-resistant cells than in mice bearing tumors derived from sunitinib-resistant cells (Fig. 4B). As expected, control cells metastasized to the lungs, but both sunitinib-resistant cells and sorafenib-resistant cells increased the lung metastasis rate compared to that in the control group (Fig. 4C). Analysis of tumor sections revealed that most of the sunitinib-resistant and sorafenib-resistant GFP-positive cells were present in the tumor core (Fig. 4D). In addition, sorafenib-resistant tumors were characterized by a higher GFP signal, which suggested that the sorafenib-resistant GFP-positive cells were more aggressive and proliferated faster than the control cells (Fig. 4D, E). Mice injected with sunitinib-resistant cells had the longest disease-free time compared to the mice in the other groups (Fig. 4F). The level of vascularization in tumors resistant to sorafenib or sunitinib was higher than that in control tumors (Fig. 4G–I and Supplementary Fig. 4A–C). Staining performed for endothelial cell (EC) markers, such as CD31 and ICAM-1, showed a stronger fluorescence signal in the sorafenib- and sunitinib-resistant tumors than in the control tumors, and ICAM-1 exhibited the strongest signal (Fig. 4G, H and Supplementary Fig. 4A). IHC CD31 staining of tumor sections showed an increase in the number of functional blood vessels for resistant tumors (Fig. 4I).

The development of the vasculature in tumors resistant to sunitinib may be regulated by increased secretion of the proangiogenic factor IL8 (Fig. 4J, K), whereas in sorafenib-resistant cells, it may be regulated by increased expression of MMP9 (Fig. 4J).

### Conditioned media (CM) from sunitinib- and sorafenib-resistant ccRCC cells disrupts endothelial cell monolayer integrity through phosphorylation of VE-cadherin

**Fig. 5:**
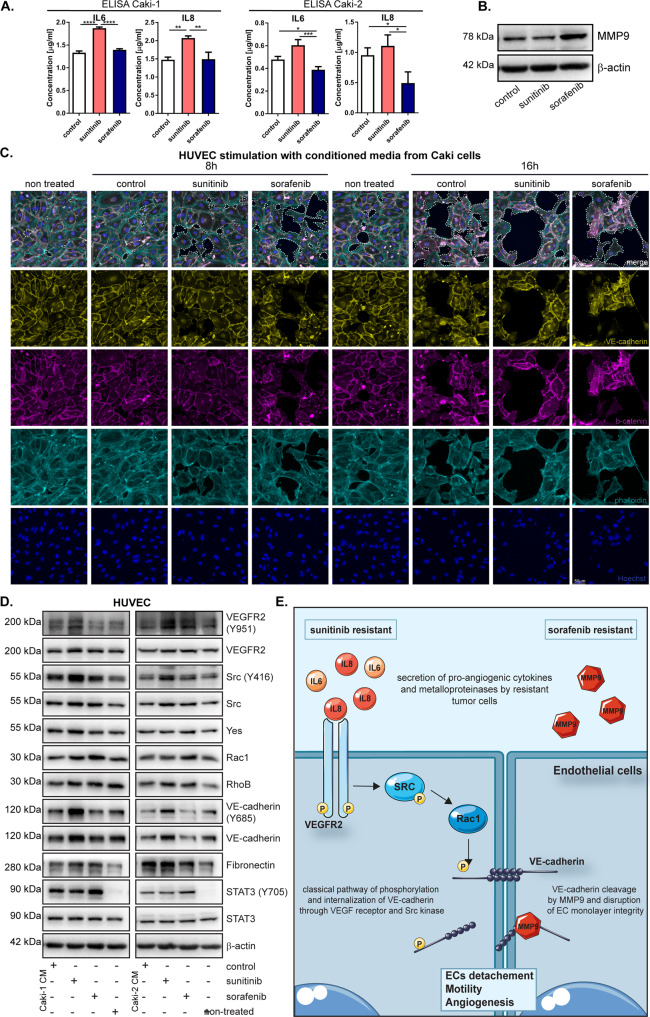
Effect of conditioned medium from drug-resistant ccRCC cells on endothelial cells activity. **A** The levels of secreted IL6 and IL8 in conditioned media from resistant Caki cells, measured by ELISA. **B** Representative western blot of MMP9 in Caki-1 cells after 7 days treatment with drugs with β-actin as a loading control. **C** Representative merged images after immunofluorescence staining of HUVEC cells after 8 or 16 h of treatment with conditioned media from Caki-2 cells, treated 7 days with sunitinib or sorafenib. Dotted lines show borders of monolayer disruption. **D** Representative Western Blot of HUVEC cells, after 3 h stimulation with conditioned media from Caki-1 or Caki-2 cell lines, treated 7 days with drugs. β-actin was used as a loading controls. **E** Schematic representation of sunitinib and sorafenib resistance. Sunitinib-resistant cells secrete high amounts of IL8 and IL6 which activates a signaling pathway from VEGFR2 through SRC, which leads to VE-cadherin internalization, loss of cell-cell contact and increased motility. Sorafenib-resistant RCC cells secrete high amount of MMP9, which cleaves VE-cadherin and leads to disruption of ECs monolayer integrity.

After weekly treatment with sunitinib, Caki-1 and Caki-2 cell lines showed significantly increased secretion of IL6 and IL8, which are important for maintaining a senescent state and angiogenesis (Fig. 5A). Sorafenib-resistant cells had an increased MMP9 protein level (Fig. 5B). There was a large disruption of monolayer integrity after treatment of human umbilical vein endothelial cells (HUVECs) with CM isolated from ccRCC cells resistant to sunitinib or sorafenib (Fig. 5C). The most prominent changes were visible in HUVECs treated with CM from sorafenib-resistant Caki-1 cells for 16 h compared to control, nontreated cells (Fig. 5C). In addition, analysis of the HUVEC migratory potential after treatment with CM from Caki-1 and Caki-2 cells showed that resistance to sunitinib and sorafenib slightly increased the speed and distance traveled by endothelial cells (Supplementary Fig. 4B, C). CM from sorafenib- or sunitinib-resistant ccRCC cells also changed the levels of markers characteristic of endothelial cell monolayer integrity in HUVECs compared to control and nontreated cells. CM isolated from sunitinib-resistant cells increased the levels of phosphorylated VEGFR2, Src, Rac-1, and VE-cadherin in HUVECs (Fig. 5D). In addition, we observed the activation of proteins involved in the rearrangement of the cytoskeleton, such as RhoB and fibronectin, as well as Yes1, a Src family tyrosine kinase, after treatment of HUVECs with CM from sunitinib- or sorafenib-resistant ccRCC cells (Fig. 5D). We also observed strong activation of the transcription factor STAT3 in HUVECs due to secretion of IL6 in CM from Caki cells. We obtained the same results in another endothelial cell line, HMEC-1, after treatment with CM isolated from resistant ccRCC cells (Supplementary Fig. 4D).

The results indicate that sunitinib- and sorafenib-resistant cells affect endothelial cells differently. Sunitinib resistance leads to the classic pathway of endothelial cell activation [26, 27] through the phosphorylation of VEGFR and Src kinase followed by the phosphorylation of VE-cadherin, which is then internalized and degraded. Sorafenib resistance leads to a loss of intercellular junctions and activation of endothelial cell migration, which may occur via VE-cadherin cleavage by metalloproteinases [28–30] (Fig. 5A–E).

### Decreased MCPIP1 expression and c-Met and IRAK1 activation result from the acquisition of sorafenib and sunitinib resistance in mouse tumors and RCC cells

**Fig. 6:**
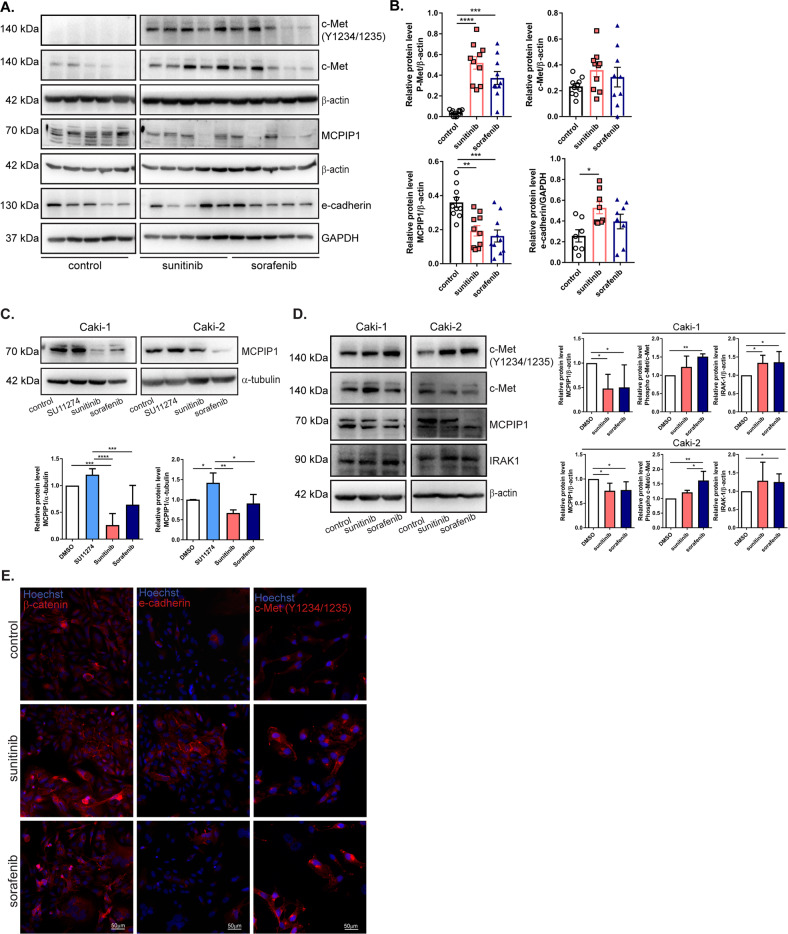
Effect of drugs treatment on MCPIP1, IRAK1, and c-Met receptor. **A** Western blot from resistant tumor specimens, collected 6 weeks after resistant RCC cell injections, with GAPDH as the loading control. *N* = 5 per group. **B** Densitometric quantification of resistant tumor samples. The results are presented as the mean ± SEM. P-Met, c-Met, MCPIP1 graphs: *N* = 10 per group; E-cadherin graph: *N* = 8 per group. **C** Representative western blot of Caki cells after 3 weeks of treatment with SU11274, sunitinib or sorafenib, with α-tubulin as a loading control. Densitometric analysis of MCPIP1 protein level. **D** Western blot of Caki cells after 24 h treatment with sunitinib or sorafenib with β-actin as a loading control. Densitometric quantification of western blot results. The results are presented as the mean ± SEM. *P* values were estimated using one-way ANOVA with post hoc Tukey’s multiple comparison test. *P < 0.05; **P < 0.01; ***P < 0.001; ****P < 0.0001. **E** Images of immunofluorescence staining of Caki-1 cells after one week of treatment with sunitinib or sorafenib. Nuclei visualized with Hoechst.

We previously documented that MCPIP1 regulates the vascularization of ccRCC tumors and influences the phosphorylation of the receptor c-Met [31]. IRAK1 phosphorylates MCPIP1, which leads to its proteasomal degradation. Moreover, it has already been shown that c-Met and IRAK1 overexpression may play significant roles in the acquisition of resistance to sunitinib and sorafenib [32]. We observed that mouse tumors formed by sunitinib- and sorafenib-resistant cells were characterized by a high level of c-Met receptor expression and a decrease in MCPIP1 expression (Fig. 6A, B). Moreover, sunitinib-resistant tumors, as expected, had a significantly increased level of E-cadherin (Fig. 6A, B). We also observed a lower level of MCPIP1 in sunitinib- and sorafenib-resistant Caki cells (three weeks of treatment) than in control cells (Fig. 6C). To determine whether c-Met phosphorylation influences MCPIP1 levels, we used the c-Met inhibitor SU11274. After inhibition of c-Met activity with SU11274, the level of MCPIP1 significantly increased (Fig. 6C).

Western blot analysis of ccRCC cells after 24 h of treatment with drugs showed that both sunitinib and sorafenib increased the phosphorylation of the c-Met receptor (Fig. 6D). Together with the upregulation of c-Met phosphorylation, we observed strong downregulation of MCPIP1 (Fig. 6D). Moreover, we found an increase in IRAK1 levels after treatment with sunitinib and sorafenib (Fig. 6D). Similar results were observed after immunofluorescence (IF) staining, together with a stronger signal of β-catenin and E-cadherin after sunitinib treatment (Fig. 6E). However, we did not observe changes in c-Met or MCPIP1 at the mRNA level (Supplementary Fig. 5A).

In addition, normal cell lines, such as HK2 and HEK293, that were treated with sunitinib and sorafenib acted similarly to RCC cells. We observed higher expression of the c-Met receptor and significant downregulation of MCPIP1 in cells treated with drugs compared to the control (Supplementary Fig. 5B, C). Crystal violet staining demonstrated that in the presence of sunitinib, cells proliferated more slowly and started to form colonies (Supplementary Fig. 5D).

MCPIP1 acts as an endonuclease and, due to its RNase activity, degrades transcripts of proinflammatory cytokines. To determine whether MCPIP1 might also regulate c-Met mRNA, we stably transduced Caki-1 cells with lentiviral vectors to overexpress MCPIP1 (pLIX-MCPIP1) or inactivate the RNase activity of MCPIP1 (pLIX D141N; Supplementary Fig. 6A, B). Our findings confirmed previous results [31], showing that the activation of MCPIP1 expression led to decreases in the phosphorylation of the c-Met receptor Src kinase and the transcription factor STAT3 (Supplementary Fig. 6A, B). Furthermore, cells carrying the point mutation D141N, which completely abolishes MCPIP1 RNase activity, showed higher levels of the c-Met receptor at both the protein and mRNA levels (Supplementary Fig. 6A–C).

### MCPIP1 overexpression partially overcomes sunitinib and sorafenib resistance

**Fig. 7:**
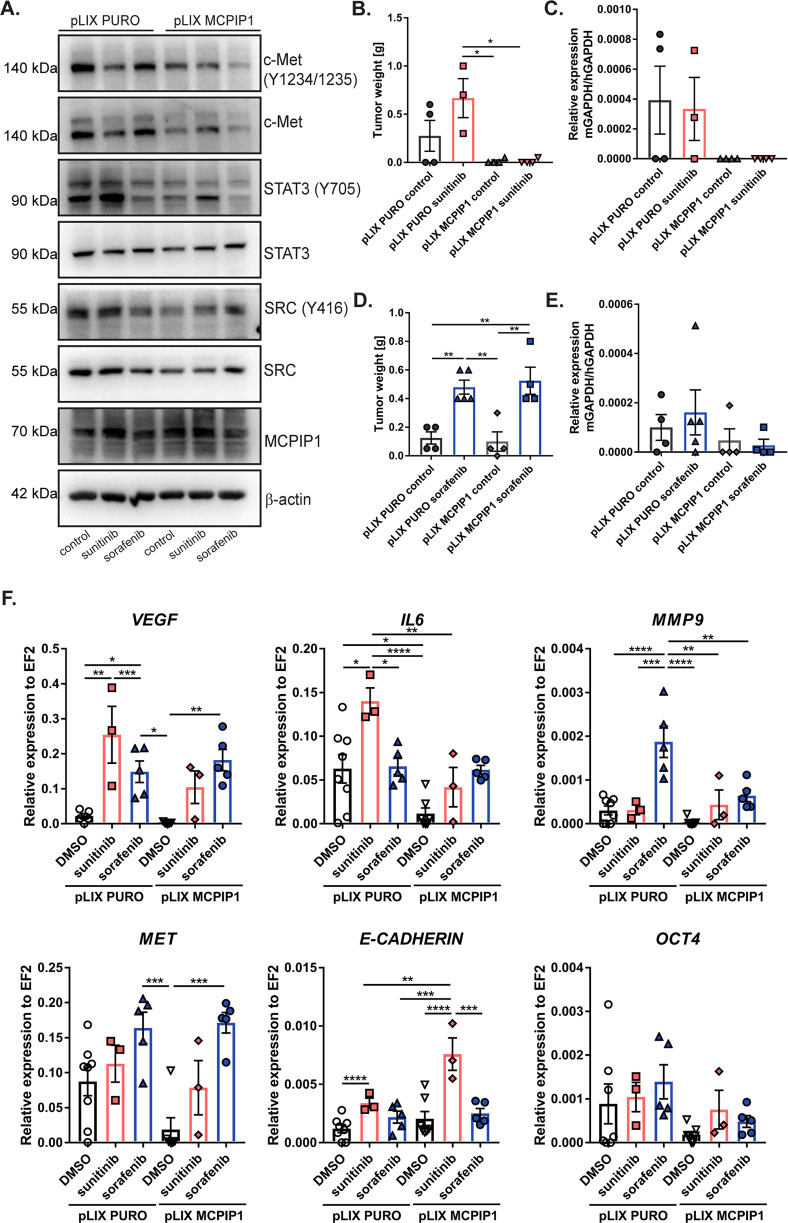
MCPIP1 in overcoming drug resistance. **A** Western blot of MCPIP1-overexpressing Caki-1 cells after 24 h treatment with drugs; β-actin was used as a loading control. **B** Effect of sunitinib resistance and MCPIP1 overexpression on tumor weight. **C** mRNA analysis of lung metastasis using real-time PCR. The results are presented as the mean ± SEM. *N* = 4 for each group except pLIX-PURO-sunitinib, where *N* = 3. **D** Effect of sorafenib resistance and MCPIP1 overexpression on tumor weight. **E** mRNA analysis of lung metastasis using real-time PCR. The results are presented as the mean ± SEM. pLIX-PURO-control, *N* = 4; pLIX-PURO-sorafenib, *N* = 5; pLIX-MCPIP1-control, *N* = 4; pLIX-MCPIP1-sorafenib, *N* = 4. **F** mRNA analysis of tumor tissue samples. The results are presented as the mean ± SEM. pLIX-PURO-control, *N* = 8; pLIX-PURO-sunitinib, *N* = 3; pLIX-PURO-sorafenib, *N* = 5; pLIX-MCPIP1-control, *N* = 8; pLIX-MCPIP1-sunitinib, *N* = 3; pLIX-MCPIP1-sorafenib, *N* = 4. In vitro experiments were performed at least three times. *P* values were estimated using one-way ANOVA with post hoc Tukey’s multiple comparison test. *P < 0.05; **P < 0.01; ***P < 0.001; ****P < 0.0001.

A decrease in the protein level of MCPIP1 leads to increased vascularity and, consequently, tumor growth and metastasis [31]. We investigated whether MCPIP1 overexpression plays a protective role and decreases the levels of factors important for migratory, vasculogenic, and invasive potential. Our results showed that a high MCPIP1 level decreased the phosphorylation of the c-Met receptor, Src kinase, and the transcription factor STAT3, even after sunitinib and sorafenib treatment (Fig. 7A). In addition, high MCPIP1 expression suppressed the growth and lung metastasis of sunitinib-resistant tumors (Fig. 7B, C). Although we did not observe a decrease in sorafenib-resistant tumor weight, the number of metastases was greatly reduced in MCPIP1-overexpressing tumors, which supported the hypothesis that MCPIP1 may at least partially protect against increased malignancy in sunitinib- and sorafenib-resistant cells and act as a tumor suppressor (Fig. 7D, E). Analysis of tumors formed from sunitinib-resistant cells revealed that the levels of *VEGF, IL6*, and *MET* decreased after MCPIP1 overexpression in control and sunitinib-resistant cells (Fig. 7F). Similar to the in vitro results, sorafenib resistance led to a strong increase in *MMP9* in tumors from sorafenib-resistant cells. However, tumors overexpressing MCPIP1 had a more than twofold decrease in *MMP9* expression (Fig. 7F). The *E-cadherin* level in sunitinib-resistant tumors strongly increased after overexpression of MCPIP1, whereas *OCT4* expression slightly decreased (not statistically significant), which may indicate that high MCPIP1 expression promotes an epithelial phenotype without stemness features (Fig. 7F).

### MCPIP1 protein expression negatively correlates with IRAK1 and c-Met expression during ccRCC tumor progression

**Fig. 8:**
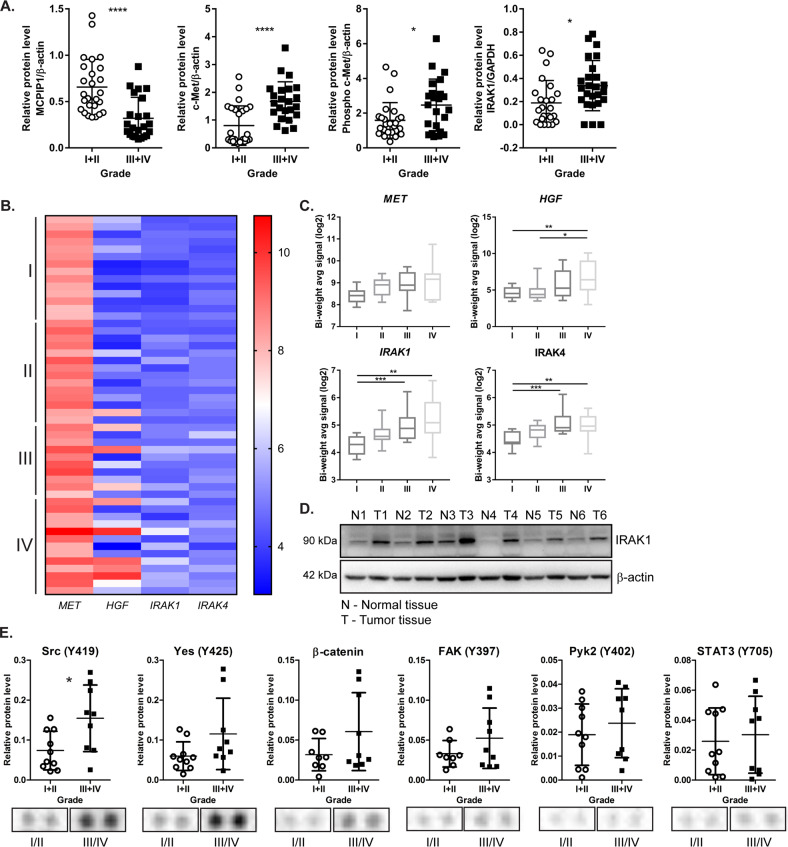
The levels of MCPIP1, IRAK1, and c-Met receptor in tumor tissue samples from ccRCC patients. **A** Quantification of the protein level of 51 tumor samples (assessed by western blot), divided into four groups according to tumor grade. **B** Heatmap showing the correlation between western blot and microarray analysis from the same patient tissue samples. Each row represents an individual tissue sample. *N* I = 14, *N* II = 14, *N* III = 10, *N* IV = 13. **C** Quantification of the signal from microarray. Statistical analysis was performed using one-way ANOVA between subjects (unpaired) where *N* = 15 per group. **D** Western blot analysis of IRAK-1 in patient tissue samples (N - normal tissue; T - tumor tissue) with β-actin as a loading control. **E** Proteome-profiler analysis from 20 patient tissue samples (*N* = 5 per each group, except group III where *N* = 4). Each dot from the arrays represents one sample from grade I/II and grade III/IV patient tumor. *P* values were estimated using two-tailed unpaired Student’s *t* test. *P < 0.05; **P < 0.01; ***P < 0.001; ****P < 0.0001.

Recently, we showed that the MCPIP1 level decreases during ccRCC progression [31]. In the present report, we show that along with the reduction in MCPIP1, the protein levels of total and phosphorylated c-Met and IRAK1 increased in RCC patient surgical specimens (Fig. 8A). These observations led us to examine the expression of *MET* and its ligand *HGF*, as well as the expression of *IRAK1* and *IRAK4*, in patient tumors; microarray gene expression analysis was used for this purpose. There was a significant increase in the expression of genes associated with ccRCC progression. We observed the highest expression in grade III and IV RCC patient specimens, which exhibited the lowest MCPIP1 protein level (Fig. 8B, C). The largest difference in IRAK1 levels was observed between normal and tumor tissues. We found that in each case analyzed, the level of IRAK1 in the tumor tissue increased drastically compared to that in the normal tissue (Fig. 8D).

Further analysis of RCC patient specimens using a protein array showed that higher levels of phosphorylated kinases, such as Src and Yes, FAK, Pyk2, β-catenin, and STAT3, were related to tumor invasiveness and metastasis, as these factors were upregulated in grade III and IV specimens versus grade I and II specimens (Fig. 8E).

## Discussion

Despite the fact that targeted therapies have changed the treatment landscape of advanced ccRCC after the cytokine era, acquired resistance is now a real problem for a large group of ccRCC patients. The present study showed that although sunitinib and sorafenib inhibit similar molecular pathways, resistance to these drugs promotes different RCC cell phenotypes. Sorafenib-resistant cells were characterized by an elongated spindle-like shape, with high migratory activity, increased expression of mesenchymal markers (*vimentin, ZEB1, SLUG*, and *TWIST*), and a high level of *MMP9*. In contrast, sunitinib resistance led to decreased expression of EMT-related genes, a lower proliferation rate, loss of cell migration ability, significantly increased E-cadherin, IL6, and IL8 expression, and a strong increase in the activity of the senescence marker SA-β-gal.

The results of the present study are in concordance with published results for liver cancer cells: these cells become spindle-shaped, lose E-cadherin expression, and gain mesenchymal markers such as vimentin when resistance to sorafenib develops [33]. In contrast to the study by Hwang et al. [34], which demonstrated a mechanism of sunitinib resistance based on EMT [34], the present results indicate an opposite effect in which sunitinib resistance decreases the expression of EMT-related genes and induces IL6 and IL8 expression and SA-β-gal activity. It has been shown that IL6 and IL8 affect growth arrest, increase SA-β-gal activity and regulate oncogene-induced cellular senescence (OIS) [19]. Moreover, resistance to VEGFR TKI treatment can lead to cellular changes resembling senescence-associated secretory phenotypes (SASPs), promoting recurrence of tumor growth and cancer progression regulated by mTOR signaling and IL6 [19, 35]. In addition, ccRCC tumors resistant to sunitinib exhibited increased IL8 expression and increased plasma levels of IL8 in mice with sunitinib-resistant tumors compared to mice bearing sunitinib-sensitive tumors [36]. Moreover, treatment with sorafenib, sunitinib, and pazopanib stimulated the autocrine secretion of IL6, which consequently led to TKI resistance in RCC cells [37]. In this study, we obtained similar results, as sunitinib resistance increased the levels of IL6 and IL8, which might activate senescence in ccRCC cells and thus increase the metastatic potential of the drug-resistant cells.

IL6 and IL8 expression is regulated at the transcriptional level by MCPIP1, which is essential for the degradation of short-lived transcripts encoding inflammation-related cytokines [38–41]. MCPIP1 also protects against the promotion of ccRCC angiogenesis via inhibition of the secretion of proangiogenic factors, such as VEGF, hypoxia-inducible factors (HIFs), IL8 and IL6 [31, 42]. In the present report, we demonstrated that both sunitinib- and sorafenib-resistant cells had reduced MCPIP1 expression, despite the different mechanisms of resistance. The level of MCPIP1 decreased significantly during prolonged exposure to sunitinib and sorafenib, which may partially explain the observations made by others that sunitinib-resistant tumors have increased hypoxia, HIF1α accumulation, and upregulation of c-Met and EMT markers such as Snail1, N-cadherin, and vimentin [43]. Our previous findings indicate that low MCPIP1 expression induces the expression of Snail1 and vimentin and increases the expression and phosphorylation of c-Met, whereas increased MCPIP1 suppresses tumor growth and inhibits the metastatic process [31].

Sunitinib resistance decreased MCPIP1 expression at the protein level, increased the secretion of IL6 and IL8, and activated VEGFR2 independent of VEGF. The results obtained by our group show that MCPIP1 downregulation in ccRCC induces the secretion of IL6 and IL8, which stimulates the development of the tumor vasculature [31]. Thus, the observed increased levels of these cytokines in tumors and in ccRCC cell lines after sunitinib treatment are, at least in part, a result of the decrease in MCPIP1. We did not observe an increased level of VEGF; however, VEGFR2 may be activated in a ligand-independent manner, leading to Src kinase activation [44], or by IL8, leading to endothelial permeability [45]. Moreover, IL6 phosphorylates VE-cadherin via a Src-dependent pathway and modulates the cell‒cell adherens junctions of endothelial cells [46]. Sorafenib-resistant RCC cells use a different mechanism of endothelial disruption than sunitinib-resistant cells. Due to their mesenchymal features, sorafenib-resistant cells secrete MMP9, which cleaves VE-cadherin and disrupts endothelial cell integrity, as shown by Kiran et al. [28, 29].

Despite different mechanisms of resistance, both sunitinib treatment and sorafenib treatment led to disruption of monolayer integrity, migration of endothelial cells, and enhancement of angiogenesis. Tumor vascularization and the high motility of sorafenib-resistant RCC cells may be the basis of the increased number of lung metastases of the resistant cells. In addition to the changes in tumor vasculature that we observed, there was a high frequency of lung metastasis by sunitinib-resistant cells, possibly due to the high invasiveness of cell clusters [47, 48]. In this study, we found that sunitinib treatment led to the formation of drug-resistant cell colonies with high levels of E-cadherin, which maintained cell‒cell adhesion; high levels of c-Met phosphorylation, which increases invasiveness; and high levels of Oct4, which regulates cancer stemness. Our results indicate that phosphorylation of c-Met in sunitinib-resistant cells leads to an increase in the stem cell marker Oct4, a decrease in P-ERK and upregulation of E-cadherin, allowing cells to maintain an undifferentiated, senescent phenotype. Interestingly, Li et al. reported that the receptor c-Met may induce a cancer stem-like phenotype due to upregulation of key stem cell markers such as Sox, Nanog, and Oct4 [49]. It has also been shown that re-expression of E-cadherin is the crucial step in the MET process responsible for promoting the stemness of cancer cells and metastatic colonization [50]. Inhibition of ERK kinase was able to completely restore E-cadherin cell‒cell junctions in Ras-transformed breast epithelial cells and prostate cancer cells [24, 25].

It has previously been shown that c-Met and IRAK1 overexpression may play significant roles in the acquisition of resistance to sunitinib and sorafenib [32, 51]. Moreover, IRAK1 phosphorylates MCPIP1, leading to its proteasomal degradation [52]. In this study, we observed that in ccRCC patient samples, the protein levels of total and phosphorylated c-Met and IRAK1 increased, while MCPIP1 levels decreased. It was previously shown that IRAK1, in the TLR/IRAK pathway, is significantly upregulated in hepatocellular carcinoma (HCC) and may promote self-renewal, tumorigenicity, and liver tumor-initiating cell marker expression. Moreover, IRAK1 inhibition sensitized HCC cells to sorafenib treatment in vitro via suppression of the apoptotic cascade [51].

This study showed that a high MCPIP1 level decreased the phosphorylation of the c-Met receptor, Src kinase, and the transcription factor STAT3 in RCC cells, even after the acquisition of sunitinib and sorafenib resistance. In addition, high MCPIP1 expression reduced tumor growth and lung metastasis in sunitinib-resistant tumors. We previously revealed that modulation of MCPIP1 activity may alter cell behavior and that an increased level of MCPIP1 in ccRCC cells decreases the levels of mesenchymal markers and increases that of E-cadherin, indicating that MCPIP1 may control the acquisition of mesenchymal features [31]. Our findings may explain our observations of suppressed progression of tumors derived from resistant cells and indicate that MCPIP1 overexpression partially overcomes the effects of sunitinib and sorafenib resistance.

In conclusion, the present study indicates separate novel mechanisms for acquired resistance to sunitinib or sorafenib in RCC cells. TKI resistance is regulated by multiple factors, including the MCPIP1 protein; this protein inhibits tumor growth and metastasis, and we postulate that it is a potential tumor suppressor. Therefore, modulation of MCPIP1 activity may alter cell behavior and could be used as a novel therapeutic strategy for patients with ccRCC.

## Supplementary information

Supplementary material

Original Data File

Editing certificate

aj-checklist

## Data Availability

The authors declare that all data supporting the findings of this study are available within the paper in the main text or the Supplementary Materials. Raw and processed data from the microarray analysis are deposited in the Gene Expression Omnibus repository: (accession number: GSE150404).
