# Supplementary material for: Resistance to tyrosine kinase inhibitors promotes renal cancer progression through MCPIP1 tumor-suppressor downregulation and c-Met activation
Source: Cell Death Dis. 2022 Sep 22;13(9):814. doi: 10.1038/s41419-022-05251-4 (PMC9500022; doi:10.1038/s41419-022-05251-4)
Supplement: Supplementary file 1 — Supplementary material [file 41419_2022_5251_MOESM1_ESM.pdf]

A.

Sunitinib Caki-1	Sunitinib Caki-2	Sorafenib Caki-1	Sorafenib Caki-2
IC50 = 4,8 uM	IC50 = 6,2 uM	IC50 = 8,2 uM	IC50 = 8,4 uM

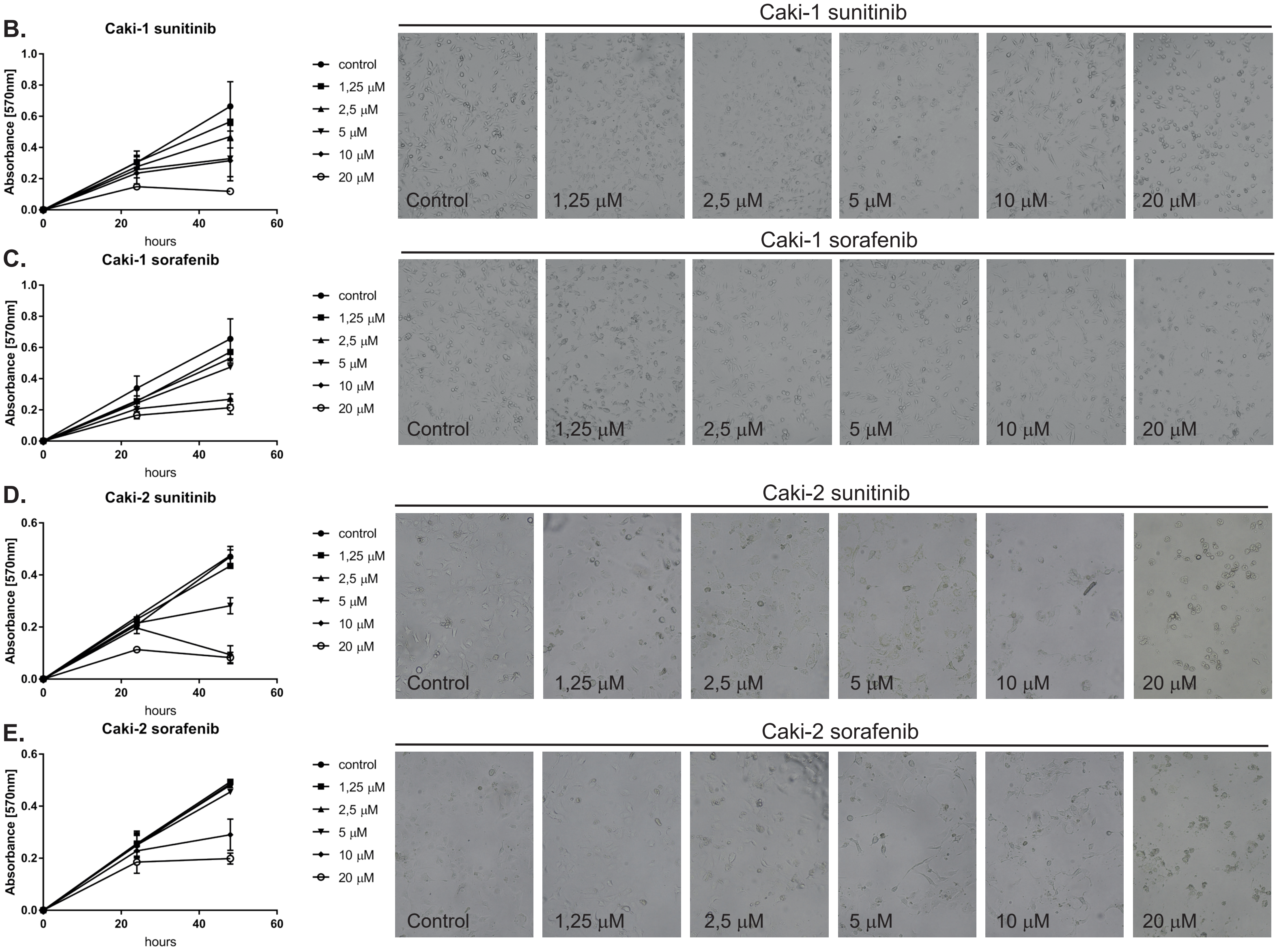

Supplementary Figure 1.

Effect of sunitinib and sorafenib on cells toxicity. A. IC50 quantification after 48h treatment with drugs. **B.** Left **-** MTT assay on Caki-1 cells treated 48 hours with sunitinib, right – representative images of cells after 48 hours treatment with different sunitinib concentrations. **C .** Left **-** MTT assay on Caki-1 cells treated 48 hours with sorafenib, right – representative images of cells after 48 hours treatment with different sorafenib concentrations. **D.** Left **-** MTT assay on Caki-2 cells treated 48 hours with sunitinib, right – representative images of cells after 48 hours treatment with different sunitinib concentrations. **E .** Left **-** MTT assay on Caki-2 cells treated 48 hours with sorafenib, right – representative images of cells after 48 hours treatment with different sorafenib concentrations. The results are presented as the triplicate means±SD of three independent experiments.

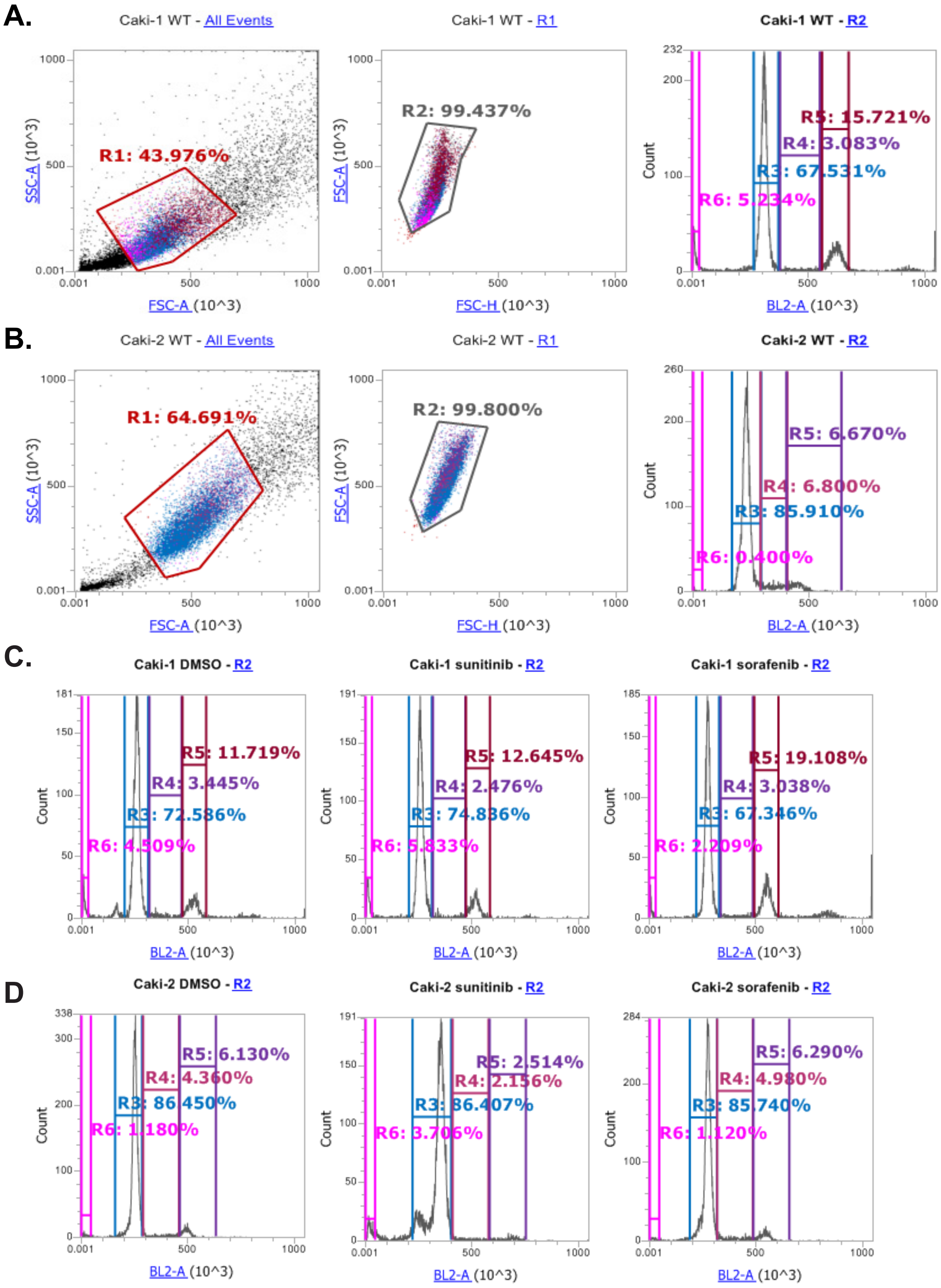
A.
Caki-1 WT - All Events
R1: 43.976%
SSC-A (10^3)
FSC-A (10^3)
Caki-1 WT - R1
R2: 99.437%
FSC-A (10^3)
FSC-H (10^3)
Caki-1 WT - R2
R5: 15.721%
R4: 3.083%
R3: 67.531%
R6: 5.234%
Count
BL2-A (10^3)
B.
Caki-2 WT - All Events
R1: 64.691%
SSC-A (10^3)
FSC-A (10^3)
Caki-2 WT - R1
R2: 99.800%
FSC-A (10^3)
FSC-H (10^3)
Caki-2 WT - R2
R5: 6.670%
R4: 6.800%
R3: 85.910%
R6: 0.400%
Count
BL2-A (10^3)
C.
Caki-1 DMSO - R2
R5: 11.719%
R4: 3.445%
R3: 72.586%
R6: 4.509%
Caki-1 sunitinib - R2
R5: 12.645%
R4: 2.476%
R3: 74.836%
R6: 5.833%
Caki-1 sorafenib - R2
R5: 19.108%
R4: 3.038%
R3: 67.346%
R6: 2.209%
Count
BL2-A (10^3)
D
Caki-2 DMSO - R2
R5: 6.130%
R4: 4.360%
R3: 86.450%
R6: 1.180%
Caki-2 sunitinib - R2
R5: 2.514%
R4: 2.156%
R3: 86.407%
R6: 3.706%
Caki-2 sorafenib - R2
R5: 6.290%
R4: 4.980%
R3: 85.740%
R6: 1.120%
Count
BL2-A (10^3)

Supplementary Figure 2.

Influence of drugs on ccRCC cell cycle. A. Flow cytometry gating of Caki-1 wild type cells after PI staining. **B.** Gating strategy of Caki-2 wild type cells after PI staining. **C.** Representative histograms with gating strategy of Caki-1 cells stimulated 7 days with drugs and stained with PI. **D.** Representative histogram graphs with gating strategy of Caki-2 cells stimulated 7 days with drugs and stained with PI.

A.

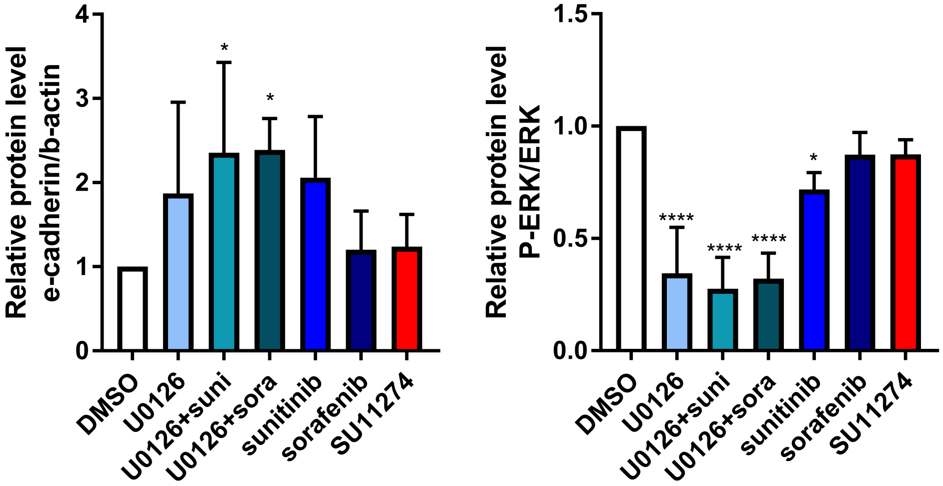

Relative protein level
e-cadherin/b-actin
4
3
2
1
0
*
*
DMSO
U0126
U0126+suni
U0126+sora
sunitinib
sorafenib
SU11274
Relative protein level
P-ERK/ERK
1.5
1.0
0.5
0.0
****
****
****
*
DMSO
U0126
U0126+suni
U0126+sora
sunitinib
sorafenib
SU11274

Supplementary Figure 3.

Effect of sunitinib, sorafenib and ERK inhibitor U0126 on Caki-1 cell line. A. Densitometric quantification of e-cadherin and phosphorylated ERK. The results are presented as the means ± SD of at least three independent experiments. P values were estimated using One-way ANOVA with *post hoc* Dunnett's test.

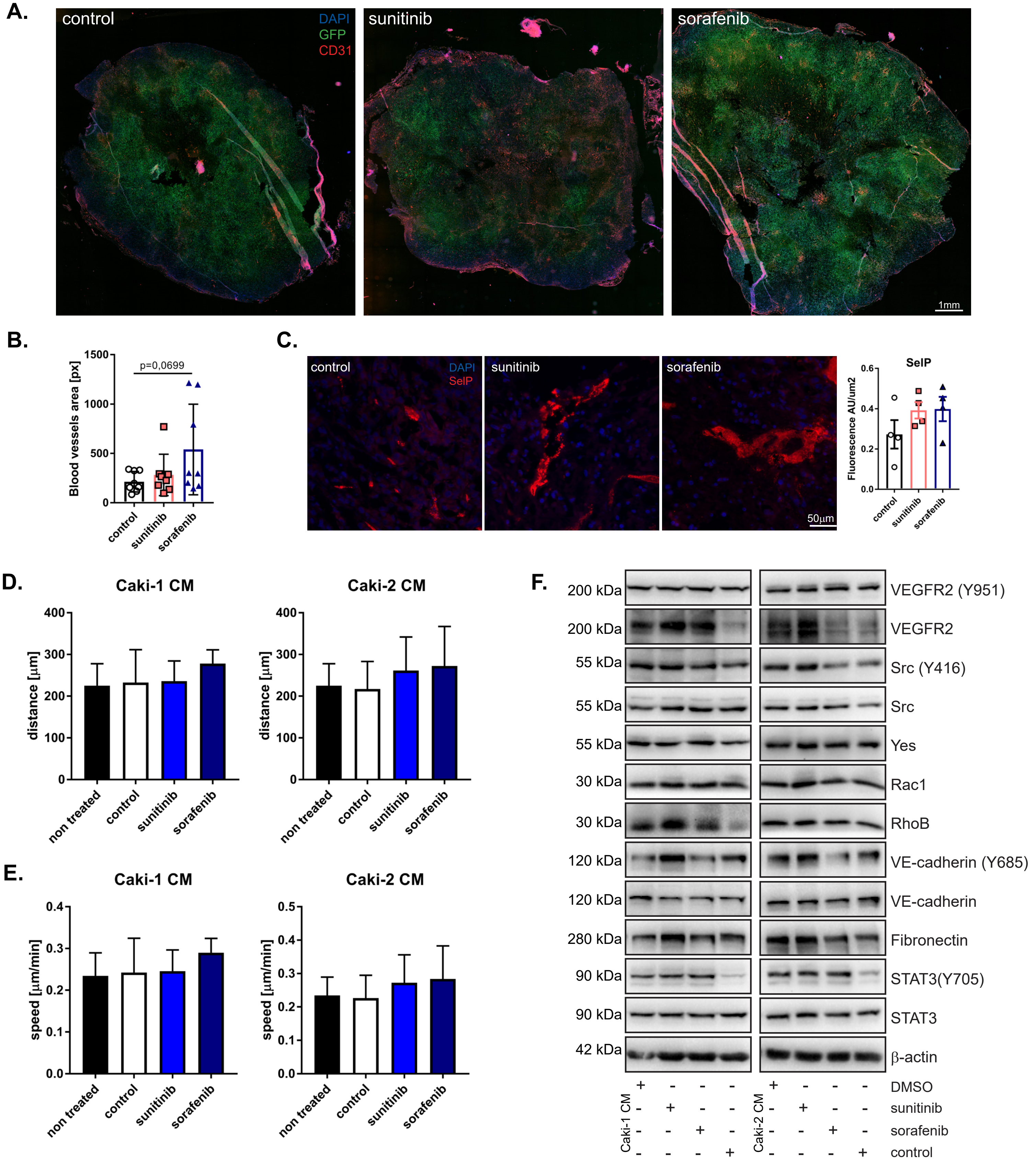

A.
control
DAPI
GFP
CD31
sunitinib
sorafenib
1mm
B.
Blood vessels area [px]
p=0,0699
1500
1000
500
0
control
sunitinib
sorafenib
C.
control
DAPI
SelP
sunitinib
sorafenib
50µm
SelP
Fluorescence AU/um2
0.6
0.4
0.2
0.0
control
sunitinib
sorafenib
D.
Caki-1 CM
Caki-2 CM
distance [µm]
400
300
200
100
0
non treated
control
sunitinib
sorafenib
E.
Caki-1 CM
Caki-2 CM
speed [µm/min]
0.4
0.3
0.2
0.1
0.0
0.5
non treated
control
sunitinib
sorafenib
F.
200 kDa VEGFR2 (Y951)
200 kDa VEGFR2
55 kDa Src (Y416)
55 kDa Src
55 kDa Yes
30 kDa Rac1
30 kDa RhoB
120 kDa VE-cadherin (Y685)
120 kDa VE-cadherin
280 kDa Fibronectin
90 kDa STAT3(Y705)
90 kDa STAT3
42 kDa β-actin
Caki-1 CM
Caki-2 CM
+ - - - + - - - DMSO
- + - - - + - - sunitinib
- - + - - - + - sorafenib
- - - + - - - + control

Supplementary Figure 4.

Role of sunitinib and sorafenib resistance on endothelial cells. A. Representative images of GFP tumor sections after immunofluorescent staining for CD31 and DAPI. **B.** Quantification of functional vessel area with a visible lumen. **C.** Representative images after immunofluorescence staining of Selectin P and quantification of Selectin P fluorescent signal. **D.** Average distance of HUVEC cells stimulated for 16 h with media from Caki-1 or Caki-2 cell lines treated with drugs for 7 days. **E.** Average speed of HUVEC cells stimulated for 16 h with media from Caki-1 or Caki-2 cell lines treated with drugs for 7 days. D and E were analyzed by measuring an individual tracks of 40 cells per group. **F.** Representative Western blot of HMEC-1 cells, after 3 hour stimulation with conditioned media from Caki-1 or Caki-2 cell lines, treated 7 days with drugs. β-actin and is as a loading control.

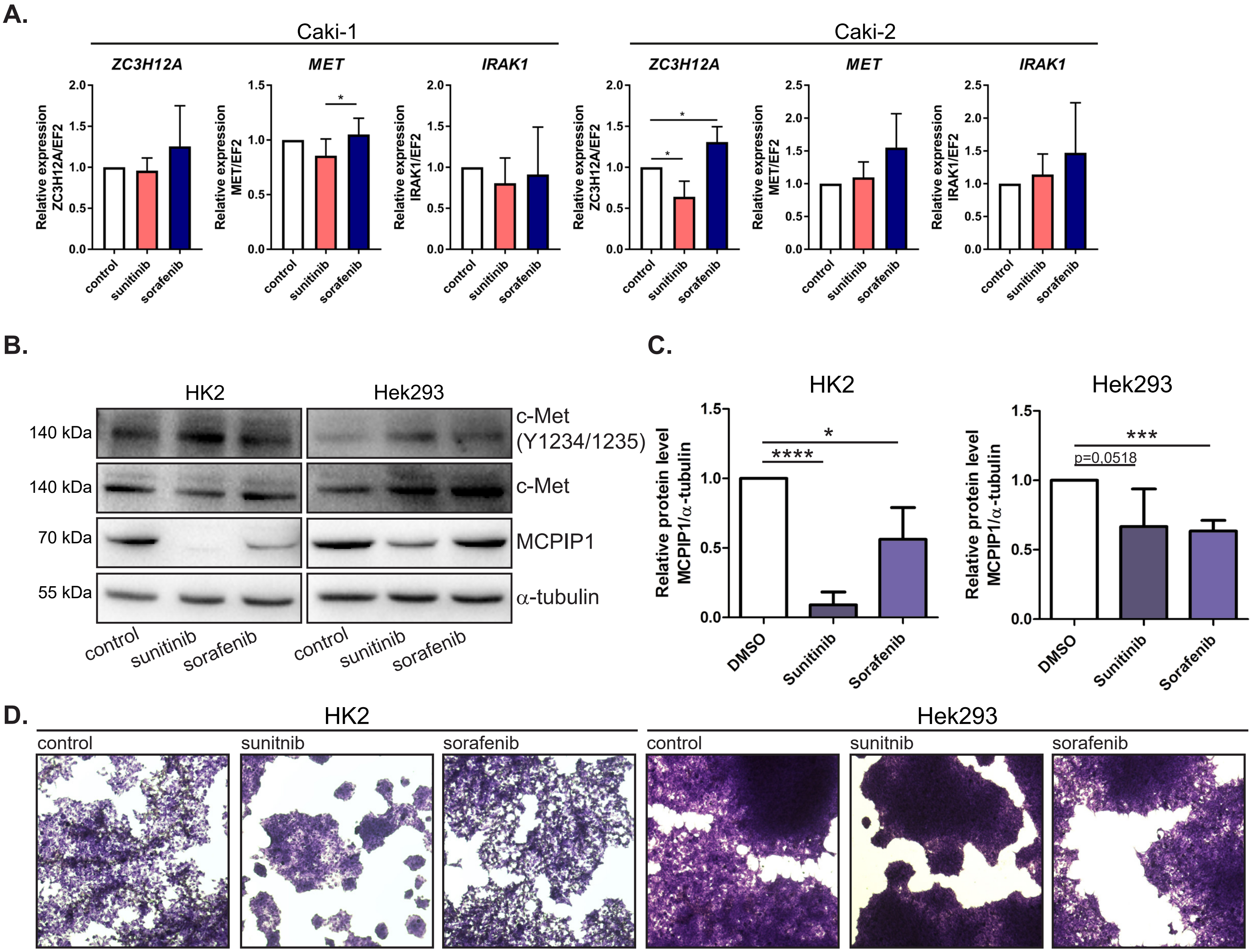

A.
Caki-1
Caki-2
ZC3H12A
MET
IRAK1
Relative expression ZC3H12A/EF2
Relative expression MET/EF2
Relative expression IRAK1/EF2
control
sunitinib
sorafenib
B.
HK2
Hek293
140 kDa
70 kDa
55 kDa
c-Met (Y1234/1235)
c-Met
MCPIP1
α-tubulin
C.
Relative protein level MCPIP1/α-tubulin
DMSO
Sunitinib
Sorafenib
p=0,0518
D.
sunitnib

Supplementary Figure 5.

Role of sunitinib and sorafenib on Caki-1 and normal epithelial cell lines HK2 and Hek293. A. Relative expression of MCPIP1, MET and IRAK1 in Caki-1 and Caki-2 cells after 24 hour stimulation with drugs, quantified with real time PCR. **B.** Representative Western blots of Hek293 and HK2 cells after 24 hour stimulation with sunitinib or sorafenib with α-tubulin as a loading control. **C.** Densitometric quantification of MCPIP1 levels assessed by Western blot. The results are presented as the means ± SD of least three independent experiments. P values were estimated using two-tailed unpaired Student t test. **D.** Crystal violet staining of HK2 and Hek293 cells after 96 hour stimulation with sunitinib or sorafenib. P-values were estimated using one-way ANOVA with post-hoc Tukey's multiple comparison test. *, $P < 0.05$; **, $P < 0.01$; ***, $P < 0.001$; ****, $P < 0.0001$.

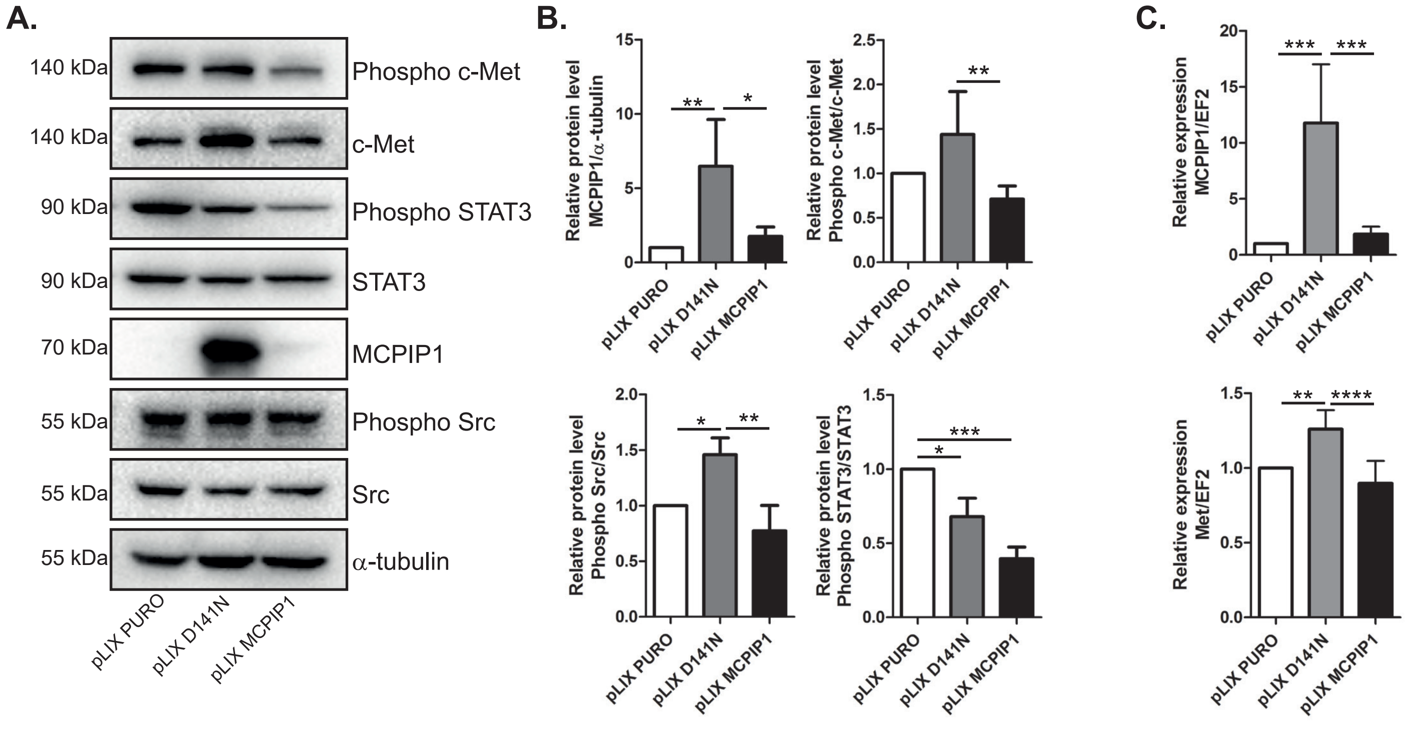
A.
140 kDa
Phospho c-Met
140 kDa
c-Met
90 kDa
Phospho STAT3
90 kDa
STAT3
70 kDa
MCPIP1
55 kDa
Phospho Src
55 kDa
Src
55 kDa
α-tubulin
pLIX PURO
pLIX D141N
pLIX MCPIP1
B.
Relative protein level MCPIP1/α-tubulin
Relative protein level Phospho c-Met/c-Met
Relative protein level Phospho Src/Src
Relative protein level Phospho STAT3/STAT3
C.
Relative expression MCPIP1/EF2
Relative expression Met/EF2

Supplementary Figure 6.

Role of MCPIP1 upregulation and inactivation on c-Met level. **A.** Representative Western blot of Caki-1 cells after upregulation (pLIX MCPIP1) or inactivation (pLIX D141N) of MCPIP1, showing levels of total or phosphorylated c-Met, STAT3 and Src kinase with α-tubulin as a loading control. **B.** Densitometric quantification of Western blots. **C.** Relative expression of MCPIP1 and c-Met transcripts in Caki-1 cells, quantified with real-time PCR. The results are presented as the means ± SD of at least three independent experiments. P values were estimated using One-way ANOVA with *post hoc* Tukey's multiple comparison test.

Gene	Sequence	Annealing temperature	Product length [nt]
**ZC3H12A**	F 5' GGAAGCAGCCGTGTCCCTATG 3'<br>R 5' TCCAGGCTGCACTGCTCACTC 3'	62 °C	226
**MET**	F 5' CATCTCAGAACGGTTCATGCC 3'<br>R 5' TGCACAATCAGGCTACTGGG 3'	62 °C	193
**MMP9**	F 5' CGCAGACATCGTCATCCAGT 3'<br>R 5' GGATTGGCCTTGGAAGATGA 3'	62 °C	406
**TWIST**	F 5' GTCCGCAGTCTTACGAGGAG 3'<br>R 5' TGGAGGACCTGGTAGAGGAA 3'	62 °C	196
**ZEB1**	F 5' CCTGTCCATATTGTGATAGAGGC 3'<br>R 5' ACCCAGACTGCGTCACATGT 3'	62 °C	195
**VIM**	F 5' TCTACGAGGAGGAGATGCGG 3'<br>R 5' GGTCAAGACGTGCCAGAGAC 3'	62 °C	213
**SLUG**	F 5' TGTTGCAGTGAGGGCAAGAA 3'<br>R 5' GACCCTGGTTGCTTCAAGGA 3'	62 °C	72
**E-CADH**	F 5' GAAGGTGACAGAGCCTCTGGAT 3'<br>R 5' GATCGGTTACCGTGATCAAAATC 3'	62 °C	122
**OCT4**	F 5' CCTTCGCAAGCCCTCATTTCA 3'<br>R 5' CCCACAGAACTCATACGGCG 3'	60 °C	267
**EF2**	F 5' GACATCACCAAGGGTGTGCAG 3'<br>R 5' TTCAGCACACTGGCATAGAGGC 3'	62 °C	215
**IL6**	F 5' GCAGAAAAAGGCAAAGAATC 3'<br>R 5' CTACATTTGCCGAAGAGC 3'	62 °C	178
**IL8**	F 5' ATGACTTCCAAGCTGGCCGTGGCT 3'<br>R 5' TCTCAGCCCTCTTCAAAAACTTCTC 3'	62 °C	292
**VEGF**	F 5' AGAAAATCCCTGTGGGCCTTGCTC 3'<br>R 5' GCCTCGGCTTGTCACATCTGCAA 3'	62 °C	155
**IRAK1**	F 5' AGCTGTCCAGGTTTCG 3'<br>R 5' CTGTACCCAGAAGGATGTC 3'	60 °C	183

Table 1 Sequences of primers, annealing temperatures and product lenght

Antibody	Dilution	Blocking buffer	Producer (cat no.)
**Mouse anti-α-tubulin**	1:1000	3 % BSA in TBST	Calbiochem (CP06)
**Rabbit anti-GAPDH**	1:1000	3 % BSA in TBST	Cell Signaling (5174)
**Mouse anti-β-actin**	1:1000	3 % BSA in TBST	Sigma (1978)
**Rabbit anti-MCPIP1**	1:1000	3 % BSA in TBST	GeneTex (gtx110807)

**Rabbit anti-Src**	1:1000	3 % BSA in TBST	Cell Signaling (2123T)
**Rabbit anti-Phospho Src (Y416)**	1:1000	3 % BSA in TBST	Cell Signaling (2101S)
**Mouse anti-β-catenin**	1:1000	3 % BSA in TBST	BD Biosciences (610154)
**Rabbit anti-e-cadherin**	1:1000	3 % BSA in TBST	Abcam (ab40772)
**Rabbit anti-c-Met**	1:1000	3 % BSA in TBST	Santa Cruz Biotechnology (sc-10)
**Rabbit anti-Phospho c-Met (Y1234/1235)**	1:1000	3 % BSA in TBST	Cell Signaling (3077S)
**Rabbit anti-STAT3**	1:1000	3 % BSA in TBST	Cell Signaling (4904S)
**Mouse anti-phospho STAT3 (Y705)**	1:1000	3 % BSA in TBST	Cell Signaling (4113S)
**Rabbit anti-VE-cadherin**	1:1000	3 % BSA in TBST	Abcam (ab33168)
**Rabbit anti-phospho VE-cadherin (Y685)**	1:1000	3 % BSA in TBST	Abcam (ab119785)
**Rabbit anti-ZO1**	1:1000	3 % BSA in TBST	GeneTex (gtx108592)
**Rabbit anti-ERK**	1:1000	3 % BSA in TBST	Cell Signaling (9102S)
**Rabbit anti-phospho-ERK (T202/Y204)**	1:1000	3 % BSA in TBST	Cell Signaling (9101)
**Rabbit anti-MMP9**	1:1000	3 % BSA in TBST	GeneTex (100458)
**Rabbit anti-VEGFR2**	1:1000	3 % BSA in TBST	Invitrogen (MA5-15157)
**Rabbit anti-phospho-VEGFR2 (Y951)**	1:1000	3 % BSA in TBST	Cell Signaling (4991T)
**Rabbit anti-Yes**	1:1000	3 % BSA in TBST	Cell Signaling (3201S)
**Mouse anti-Rac1**	1:1000	3 % BSA in TBST	Abcam (ab33186)
**Rabbit anti-RhoB**	1:1000	3 % BSA in TBST	Cell Signaling (2098S)
**Rabbit anti-IRAK1 (Caki-1 cells, tissues)**	1:1000	3 % BSA in TBST	Cell Signaling (4504)
**Goat anti-IRAK1 (Caki-2 cells)**	1:500	3 % BSA in TBST	Santa Cruz (sc-1893)
**Goat anti-rabbit IgG-HRP**	1:4000	3 % BSA in TBST	Santa Cruz Biotechnology
**Goat anti-mouse IgG-HRP**	1:4000	3 % BSA in TBST	Santa Cruz Biotechnology
**Rat anti-mouse CD31**	1:50	1% BSA in PBS	BD Pharmingen (4163758)
**Rat IgG2α κ Isotype Control**	1:50	1% BSA in PBS	BD Pharmingen (4234535)

Table 2 List of antibodies with dilutions and blocking buffers

Supplementary methods

Cell culture

The HEK-293 cell line was obtained from ATCC (cat no. CRL-1573). HK2 cells were kindly provided by the Department of Medical Biotechnology Jagiellonian University in 2015. The HEK-293 and HK2 cell lines were cultured in high-glucose Dulbecco's modified Eagle's medium (DMEM; Lonza) supplemented with 10% FBS. Human microvascular endothelial cells (HMEC-1) were obtained from ATCC (cat no. CRL-3243). HMEC-1 cells were cultured in MCDB 131 medium (Lonza) supplemented with 10% FBS, L-glutamine (2 mmol/L), epidermal growth factor (EGF) (10 ng/ml), and hydrocortisone (1 μg/ml) from Sigma-Aldrich.
