# Supplementary material for: Resistance to tyrosine kinase inhibitors promotes renal cancer progression through MCPIP1 tumor-suppressor downregulation and c-Met activation
Source: Cell Death Dis. 2022 Sep 22;13(9):814. doi: 10.1038/s41419-022-05251-4 (PMC9500022; doi:10.1038/s41419-022-05251-4)

Western blots Fig. 3D

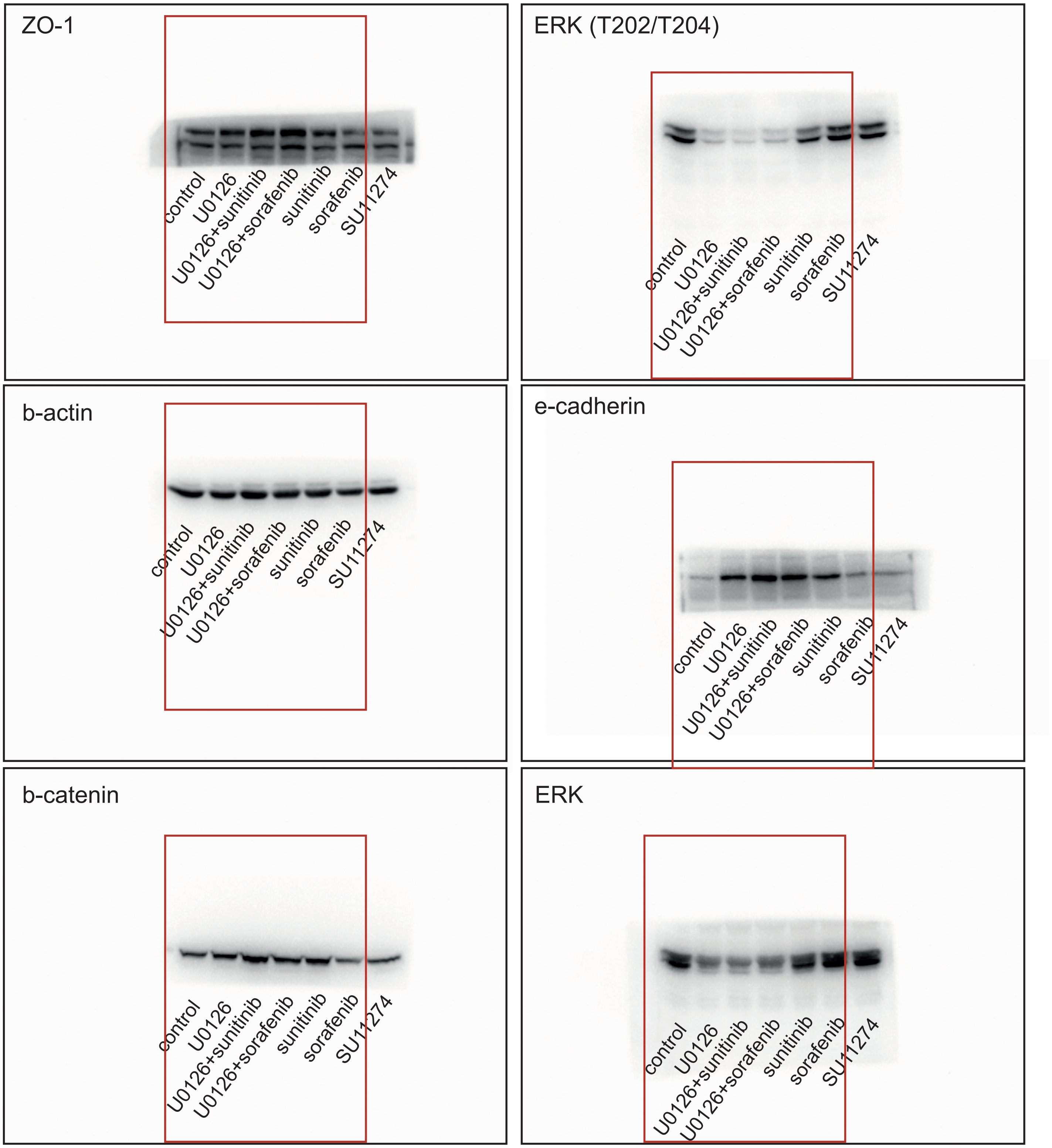

Western blots Fig. 5B

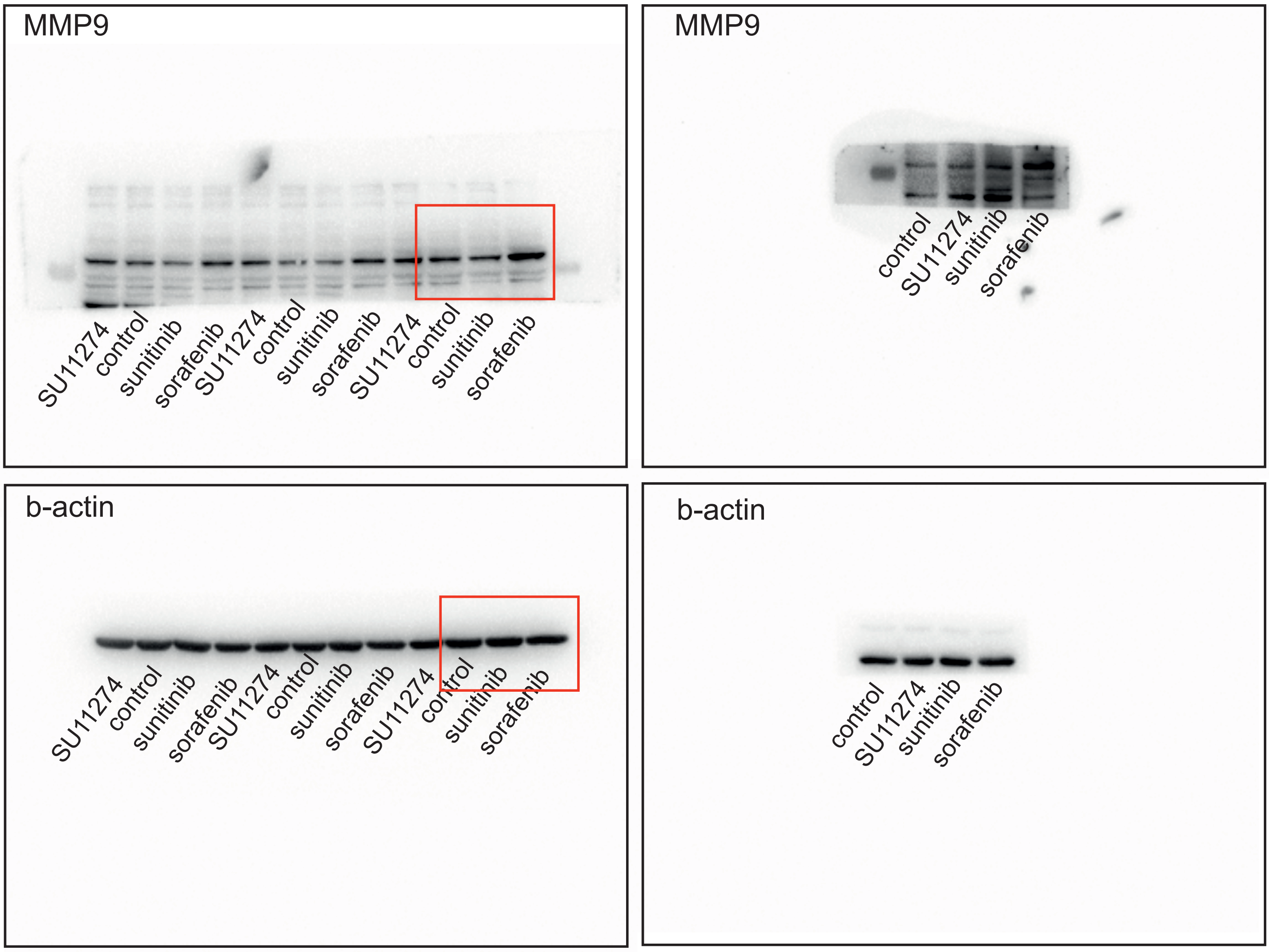

Western blots Fig. 5D

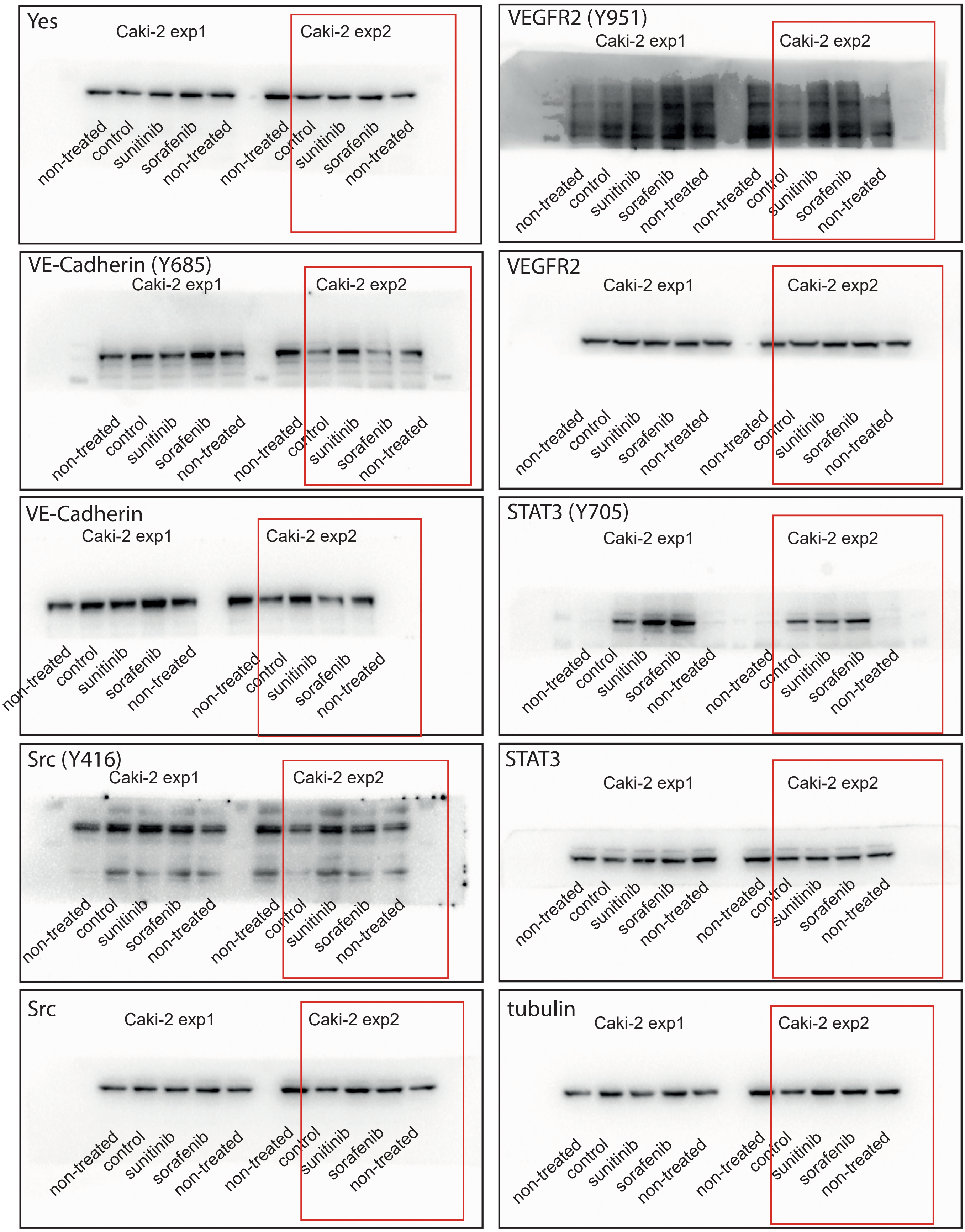

Western blots Fig. 5D

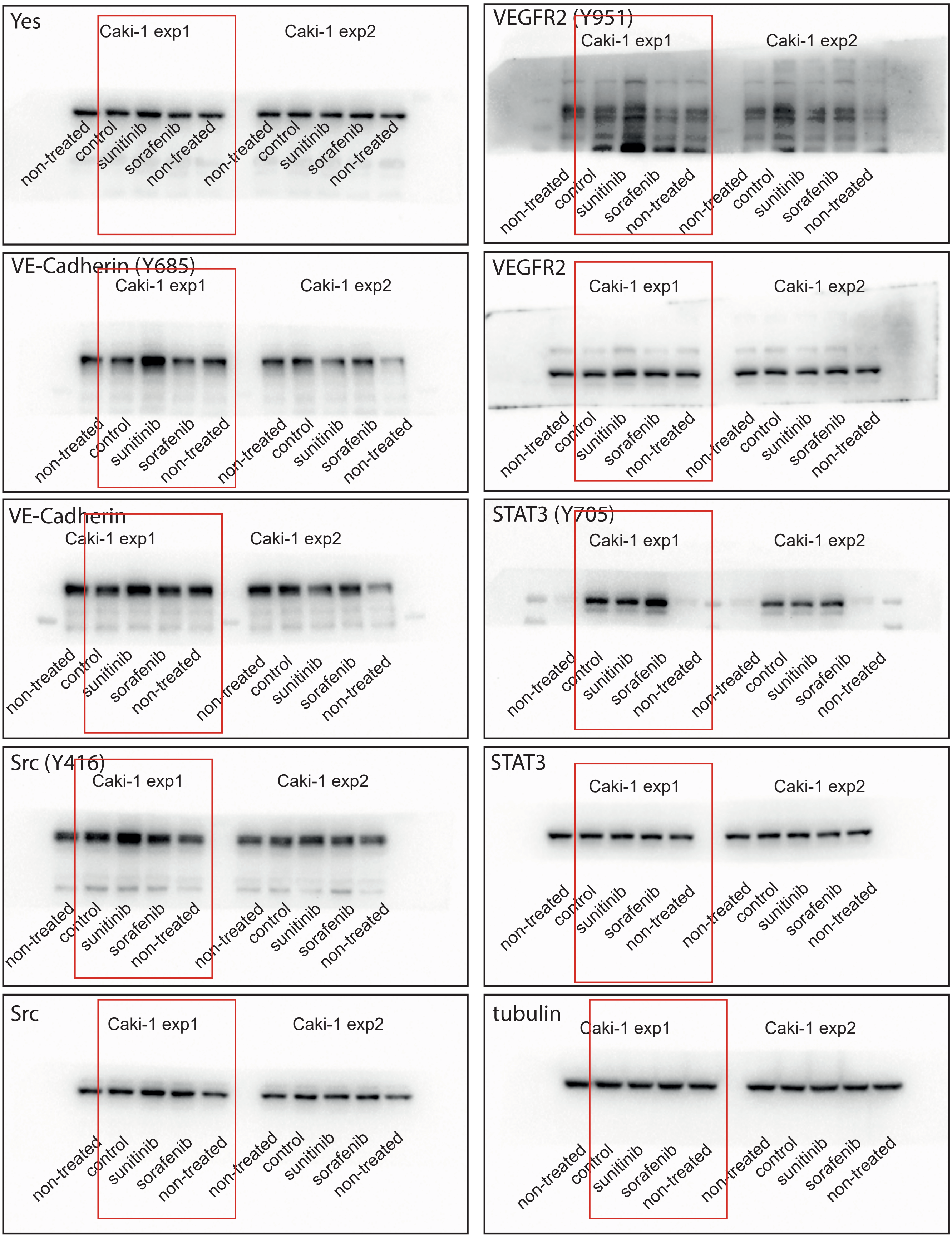

Western blots Fig. 5D

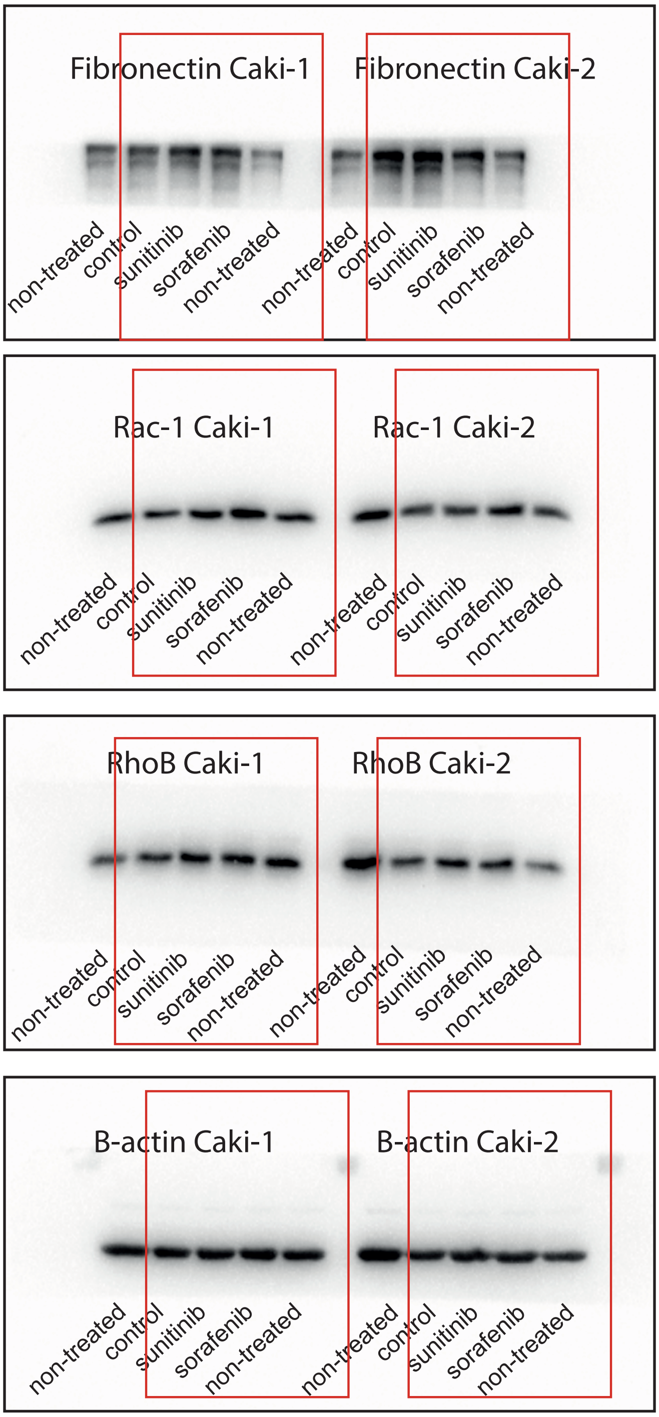

Western blots Fig. 6A

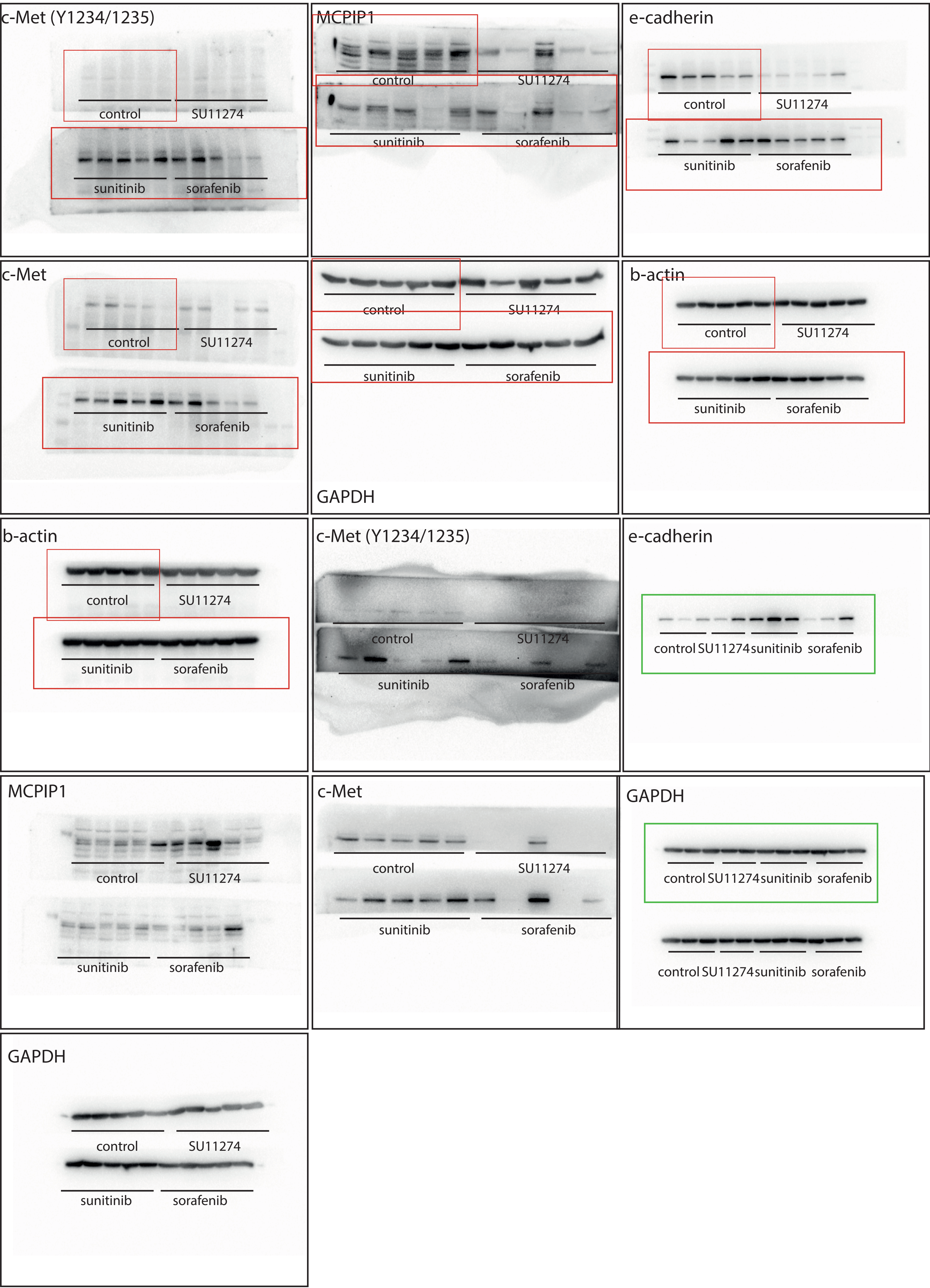

Western blots Fig. 6C

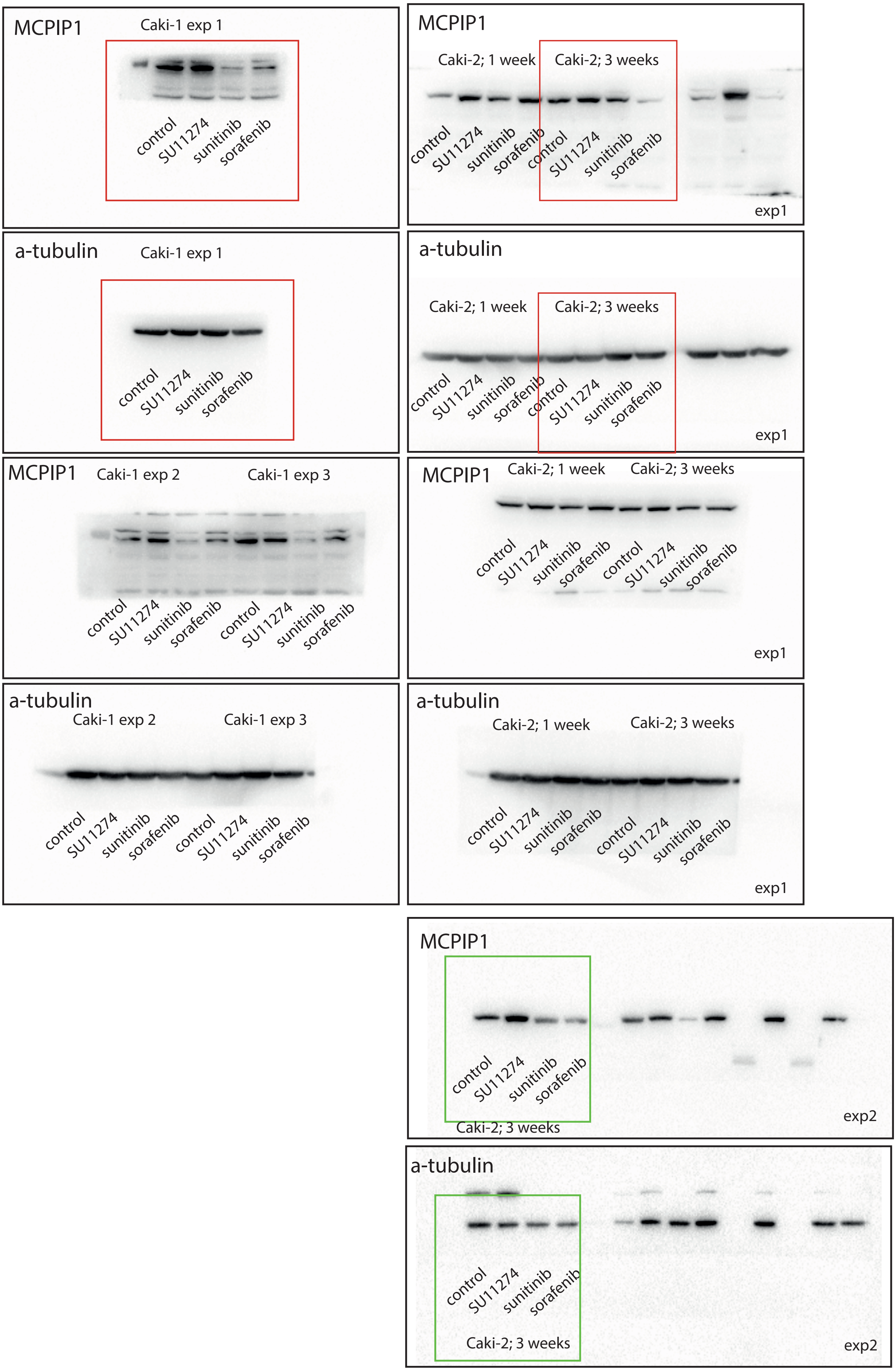

Western blots Fig. 6D

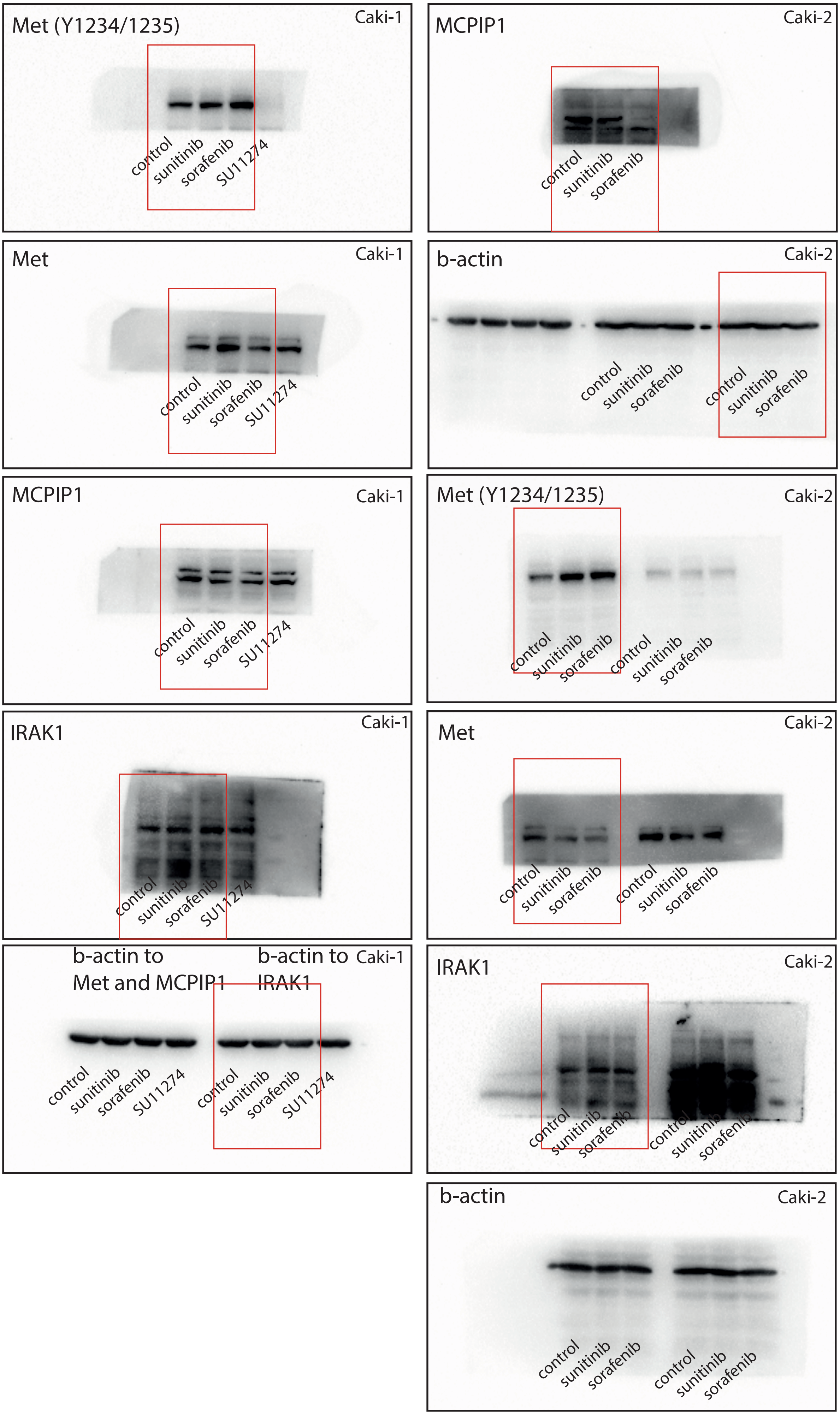

Western blots Fig. 6D

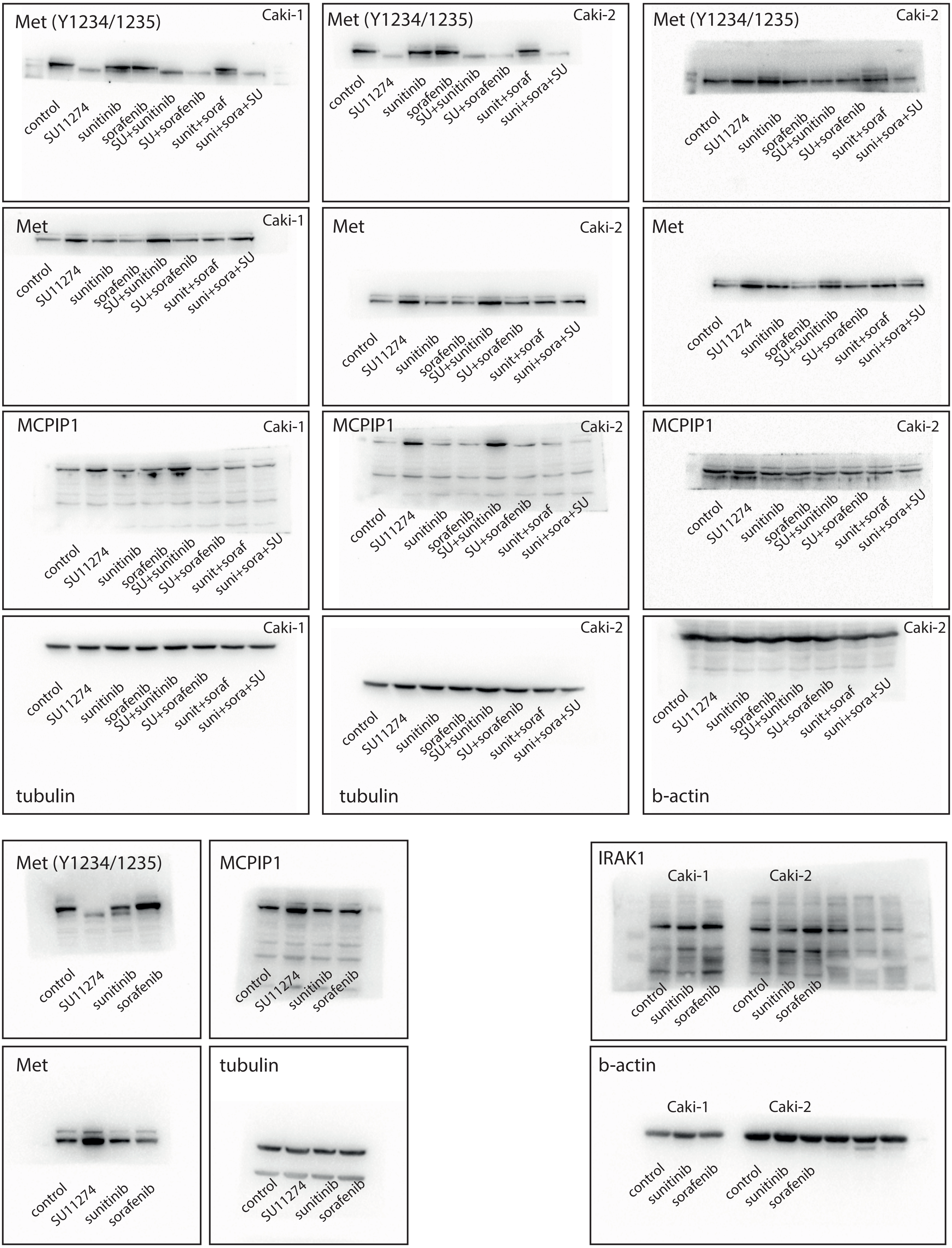

Western blots Fig. 7A

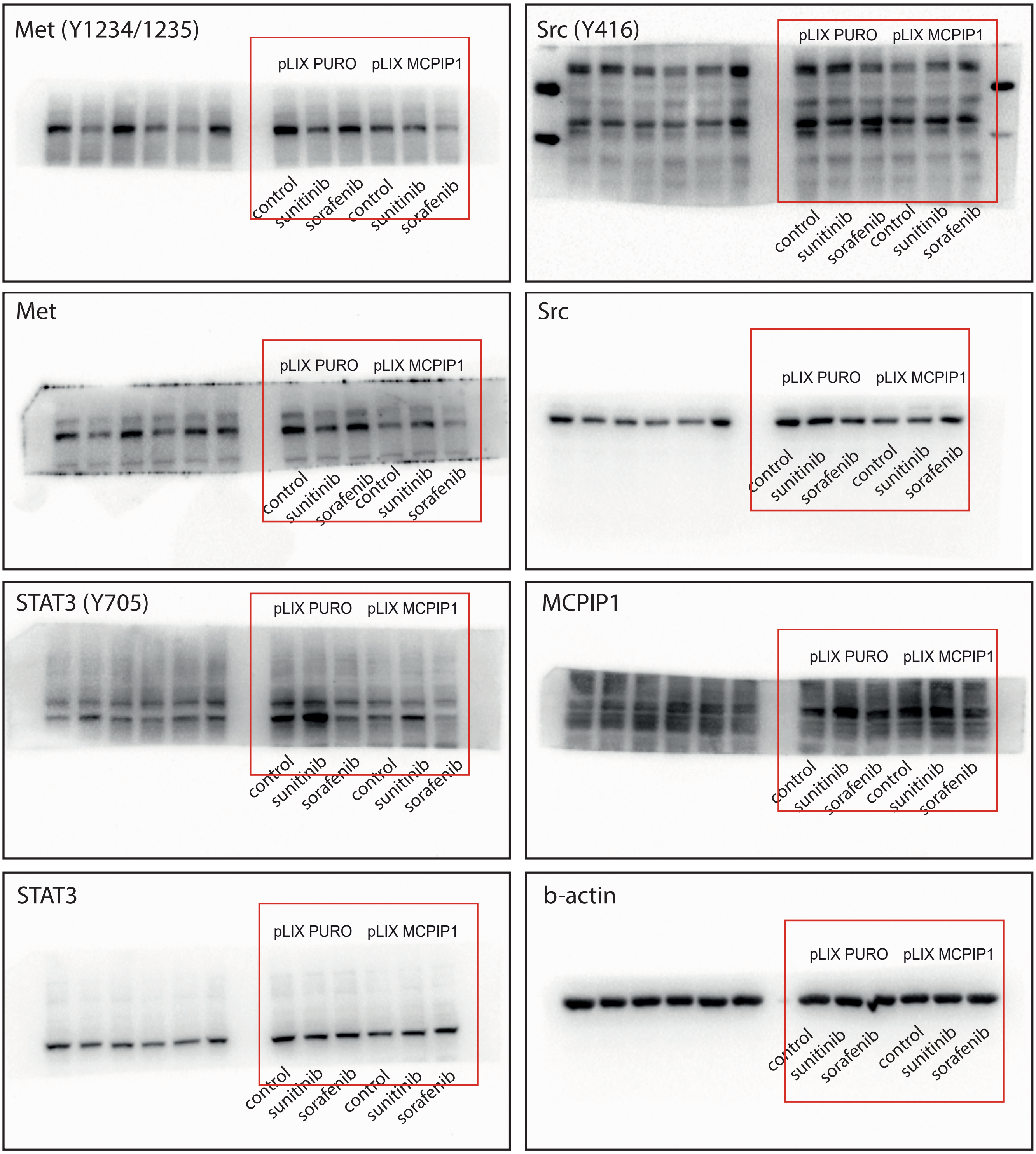

Western blots Fig. 7A

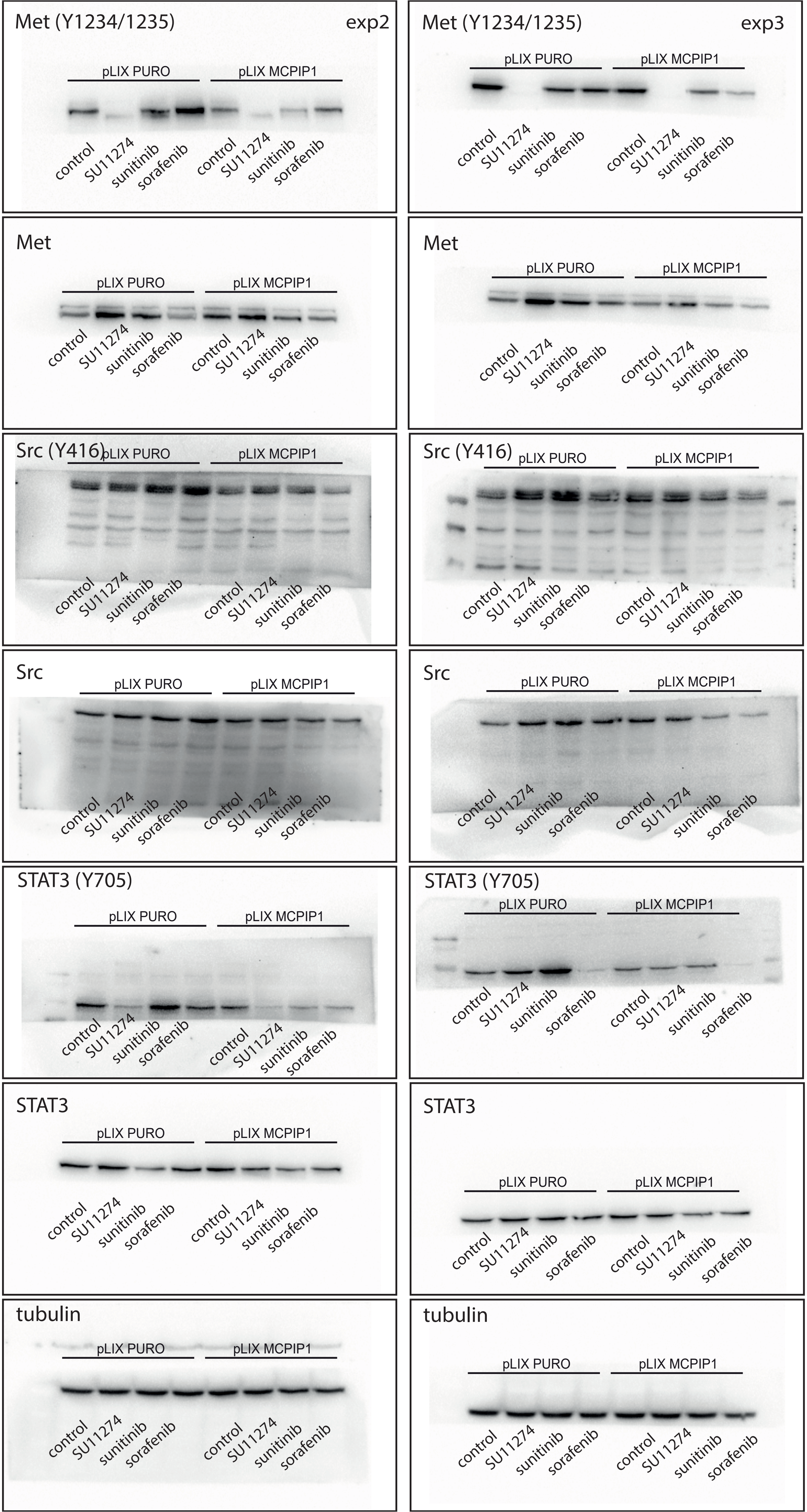

Western blots Fig. 8A

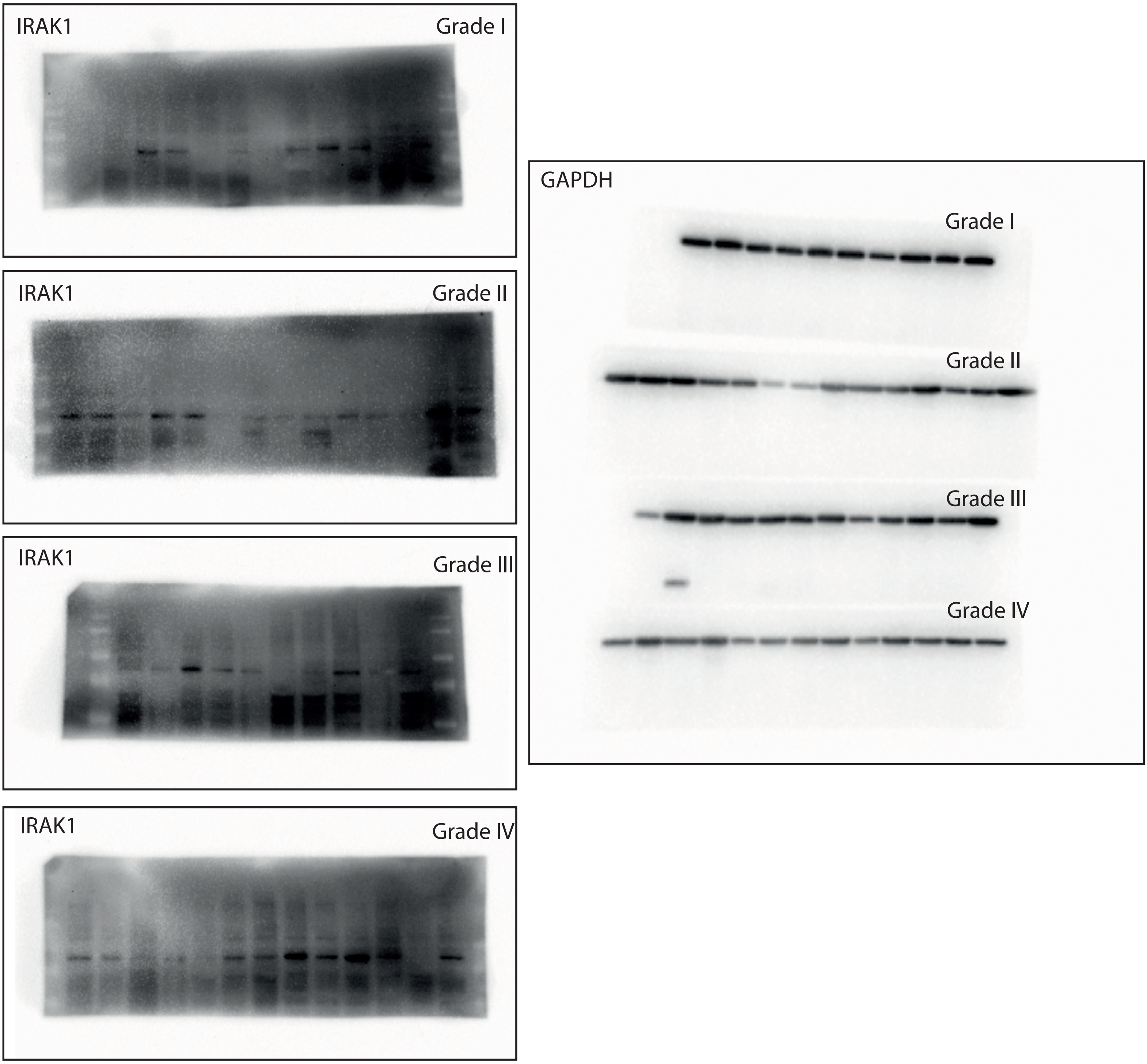

Western blots Fig. 8A

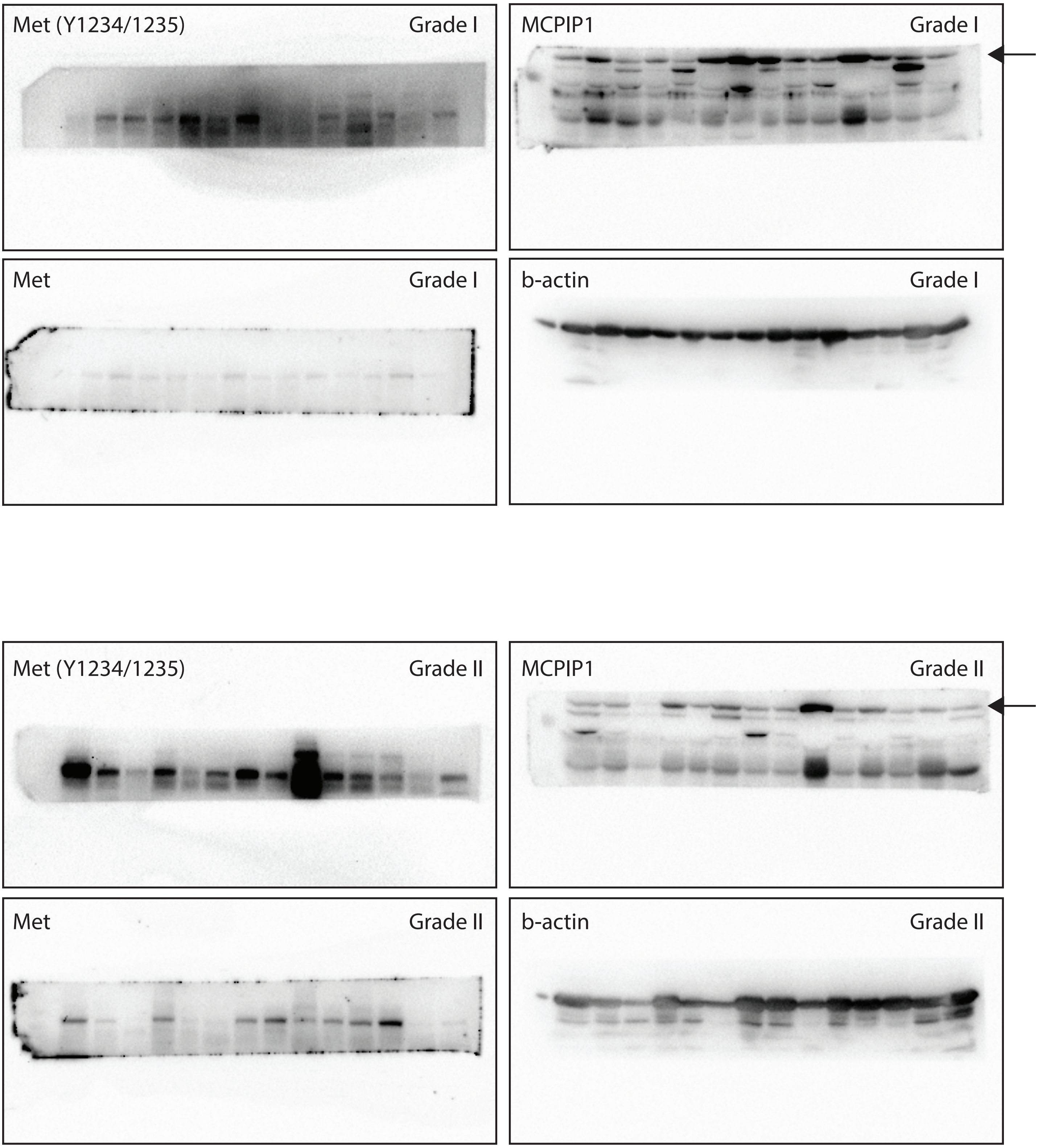

Western blots Fig. 8A

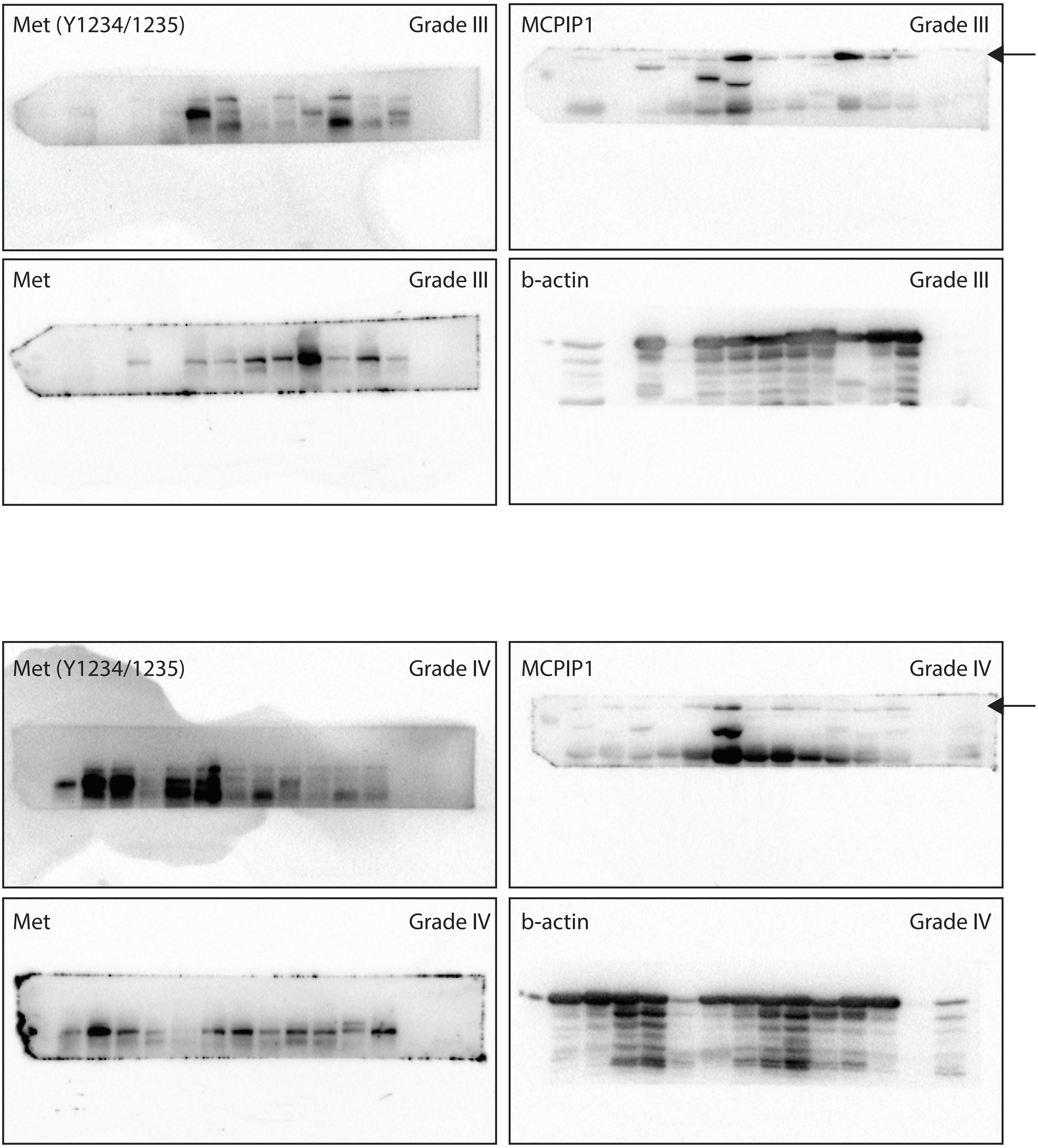

Western blots Fig. 8D

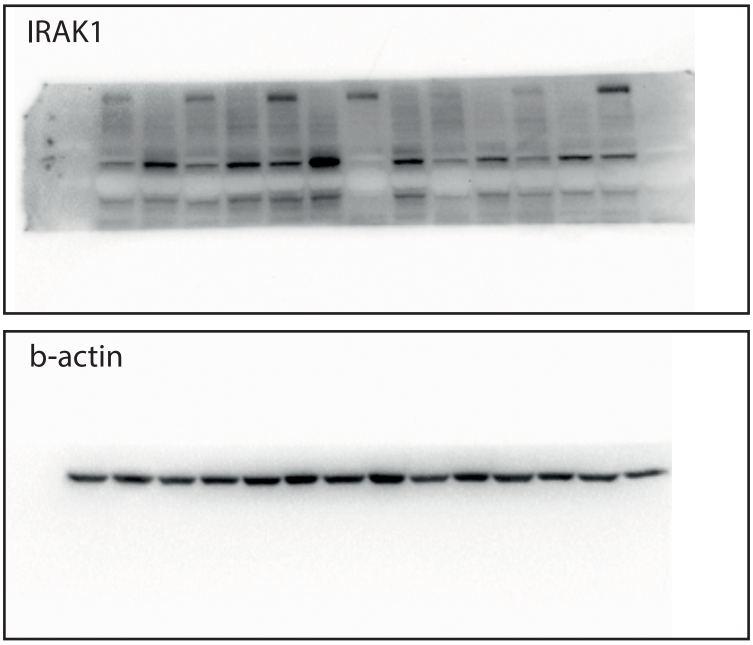

Western blots Supplementary Fig. 4D

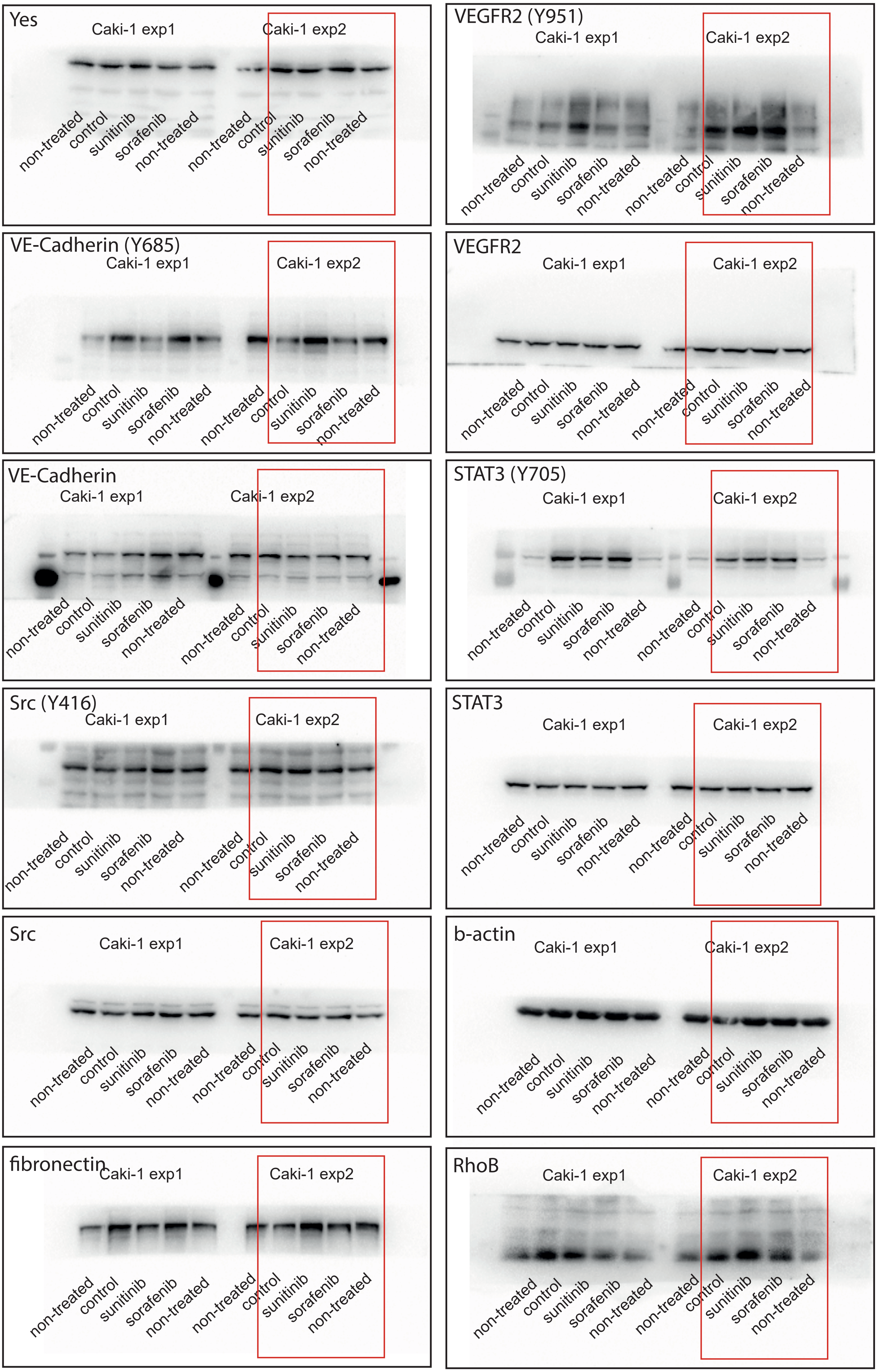

Western blots Supplementary Fig. 4D

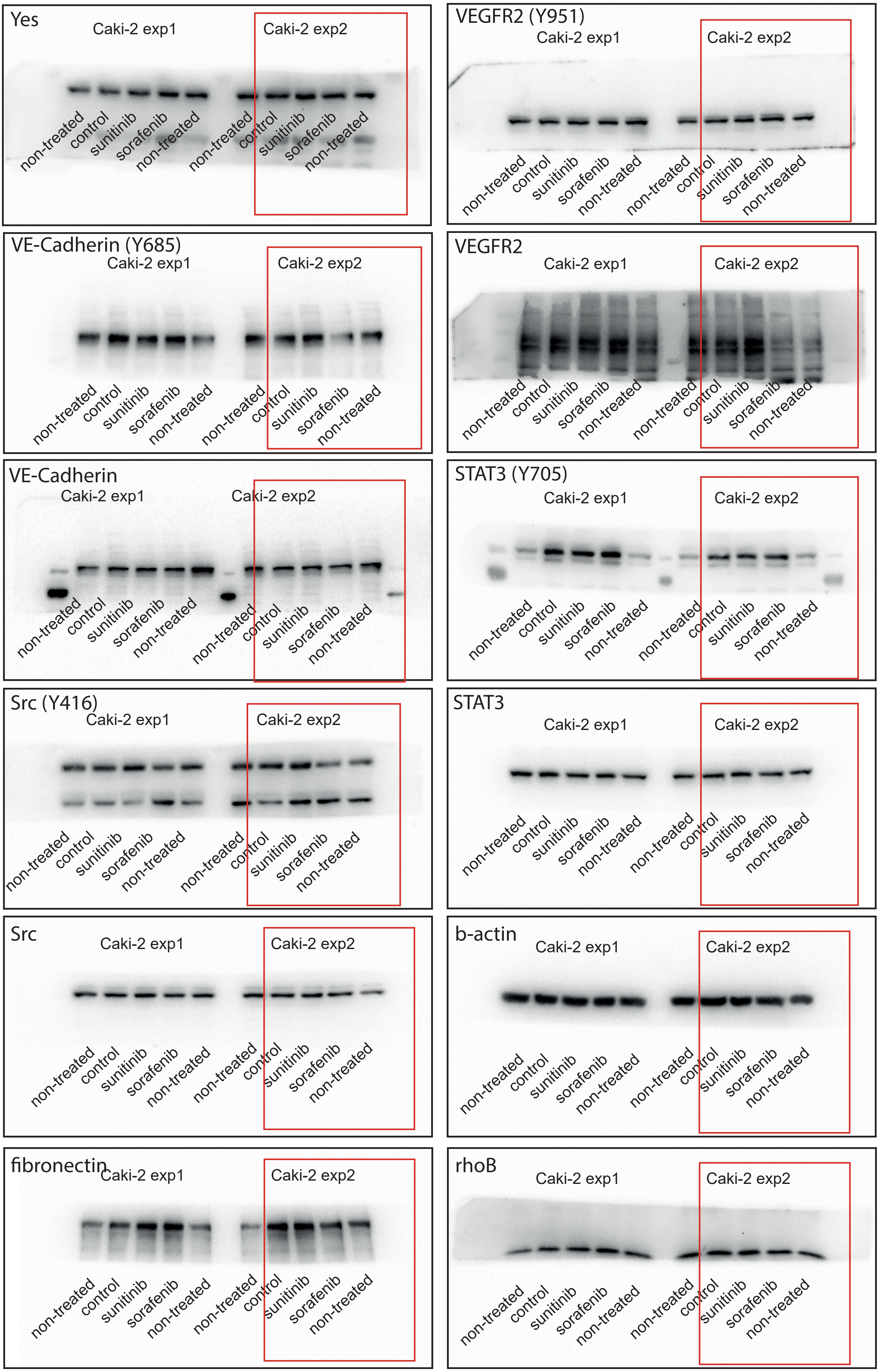

Western blots Supplementary Fig. 5B

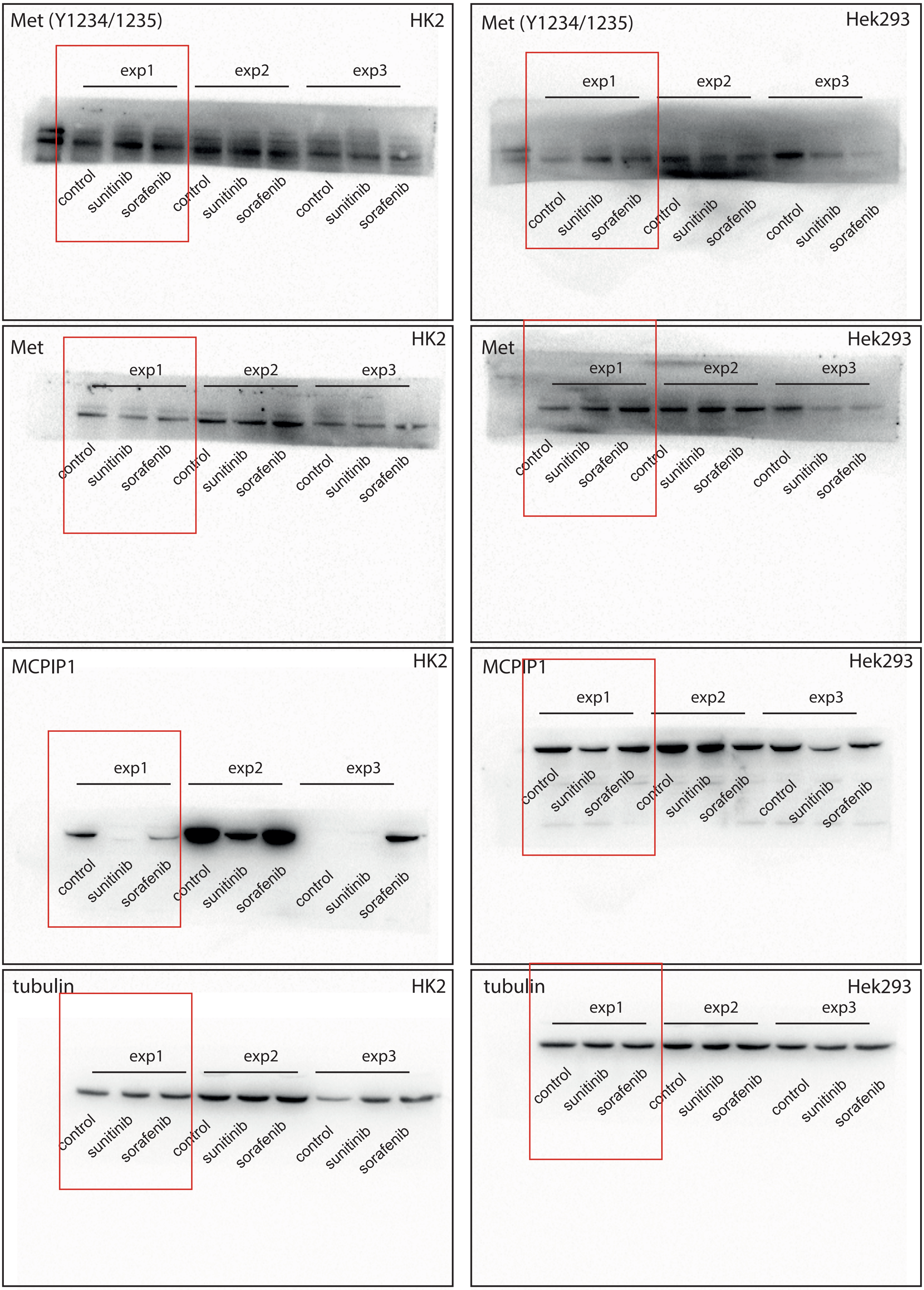

Western blots Supplementary Fig. 6

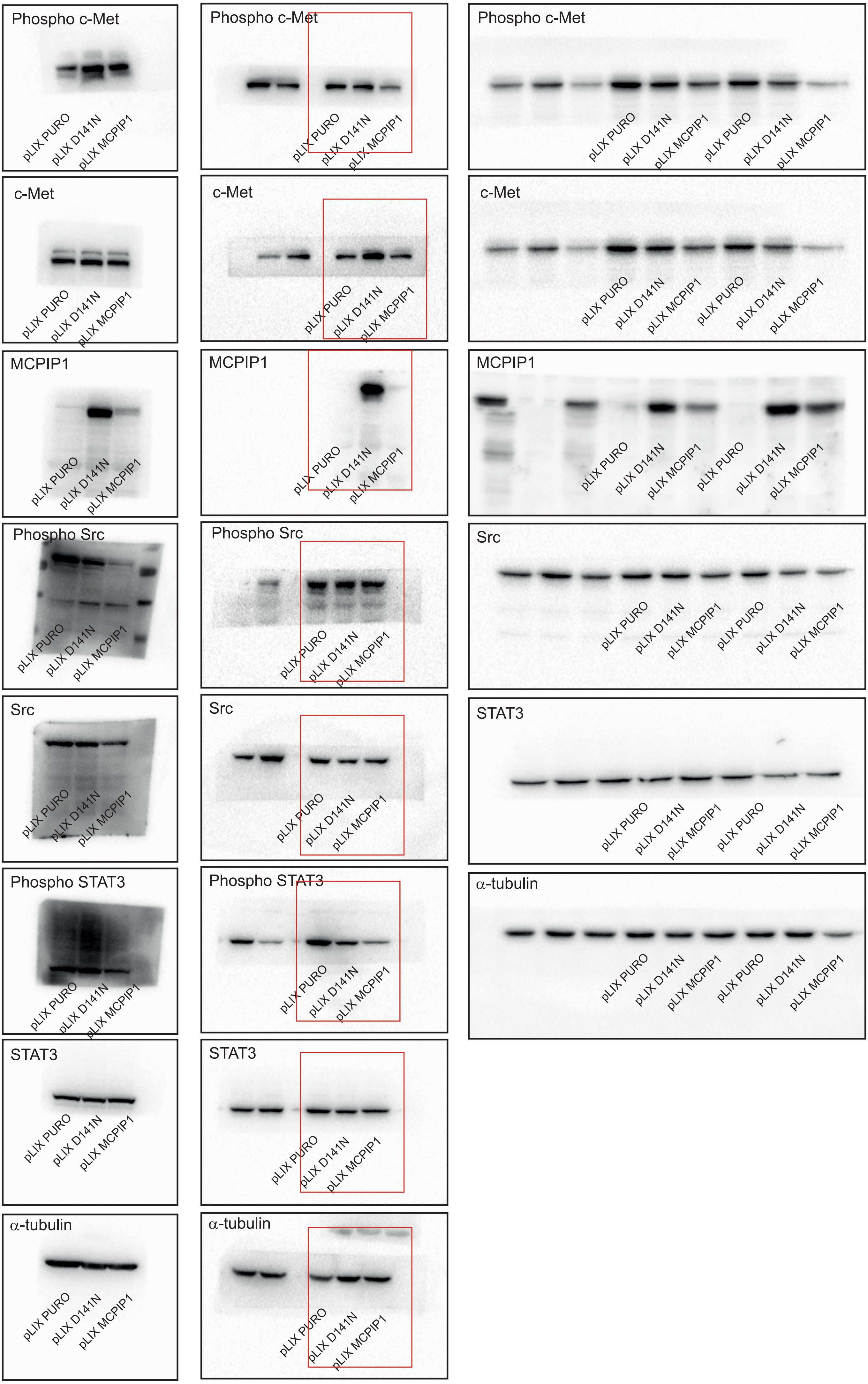

Supplement: Supplementary file 2 — Original Data File [file 41419_2022_5251_MOESM2_ESM.pdf]
