# Supplementary material for: Resistance to tyrosine kinase inhibitors promotes renal cancer progression through MCPIP1 tumor-suppressor downregulation and c-Met activation
Source: Cell Death Dis. 2022 Sep 22;13(9):814. doi: 10.1038/s41419-022-05251-4 (PMC9500022; doi:10.1038/s41419-022-05251-4)
Supplement: Supplementary file 3 — Editing certificate [file 41419_2022_5251_MOESM3_ESM.pdf]

SPRINGER NATURE

Author Services

Editing Certificate

This document certifies that the manuscript

Resistance to tyrosine kinase inhibitors promotes renal cancer progression through MCPIP1 tumor-suppressor downregulation and c-Met activation

prepared by the authors

Paulina Marona, Judyta Gorka, Oliwia Kwapisz, Janusz Rys, Jolanta Jura, Robert M Hoffman, Katarzyna Miekus

was edited for proper English language, grammar, punctuation, spelling, and overall style by one or more of the highly qualified native English speaking editors at SNAS.

This certificate was issued on **August 12, 2022** and may be verified on the SNAS website using the verification code **D713-1D17-A1E1-6B23-22CP**.

Neither the research content nor the authors' intentions were altered in any way during the editing process. Documents receiving this certification should be English-ready for publication; however, the author has the ability to accept or reject our suggestions and changes. To verify the final SNAS edited version, please visit our verification page at secure.authorservices.springernature.com/certificate/verify.
If you have any questions or concerns about this edited document, please contact SNAS at support@as.springernature.com.

SNAS provides a range of editing, translation, and manuscript services for researchers and publishers around the world.
For more information about our company, services, and partner discounts, please visit authorservices.springernature.com.
